# Donor-π-Acceptor-Type Fluorinated Tolane Containing a Semifluoroalkoxy Chain as a Condensed-Phase Luminophore

**DOI:** 10.3390/molecules28062764

**Published:** 2023-03-19

**Authors:** Shigeyuki Yamada, Keigo Yoshida, Mitsuki Kataoka, Mitsuo Hara, Tsutomu Konno

**Affiliations:** 1 Faculty of Molecular Chemistry and Engineering, Kyoto Institute of Technology, Matsugasaki, Sakyo-ku, Kyoto 606-8585, Japan; 2 Department of Molecular and Macromolecular Chemistry, Graduate School of Engineering, Nagoya University, Furo-cho, Chikusa-ku, Nagoya, Aichi 464-8603, Japan

**Keywords:** fluorinated tolane, photoluminescence, π-conjugated mesogen, donor-π-acceptor, semifluoroalkoxy flexible chain, smectic liquid crystals, phase transition, condensed phase

## Abstract

Photoluminescent liquid-crystalline (PLLC) molecules, which can easily tune the PL behavior through the crystal (Cry)–LC phase transition, have attracted significant attention. Previously, we have demonstrated that the incorporation of a semifluoroalkoxy chain into π-conjugated mesogen is a promising approach for developing PLLC molecules with PL and SmA LC characteristics. We focused on the LC and PL characteristics of the molecules induced by the semifluoroalkoxy chain and fluorinated tolanes in the condensed phase. In this study, we developed cyano- or ethoxycarbonyl-terminated donor-π-acceptor-type fluorinated tolanes containing a semifluoroalkoxy flexible chain. The cyano-terminated fluorinated tolanes exhibited intense light-blue photoluminescence in the crystalline phase and did not exhibit any LC phase. In contrast, blue photoluminescence in the ethoxycarbonyl-terminated analogs was slightly weak; however, they exhibited Cry–SmA phase transition during the heating and cooling processes. The PL intensity of the ethoxycarbonyl-terminated fluorinated tolanes significantly decreased in the SmA phase; however, their PL colors changed during the Cry–SmA phase transition. This indicates that the developed tolanes are promising temperature-dependent PL materials, such as PL thermosensors or PL thermometers.

## 1. Introduction

Precise control of molecular arrangements in the condensed phase is of great importance for the development of functional molecules because their physical properties, such as luminescence, electrical conductivity, and magnetic properties, vary significantly with the molecular arrangement [1,2,3,4]. Crystal–crystal (Cry–Cry) phase transition using polymorphs is one of the approaches to control the aggregated structures. It has been reported that luminescent molecules significantly switch through phase transitions of polymorphs [5,6]. As an alternative approach, Cry–liquid crystal (LC) or LC–LC phase transition is effective in reversibly ordering molecular arrangements because the LC phase is a mesophase between the Cry and isotropic (Iso) phases and can form different molecular arrangements depending on the LC phase [7,8]. Accordingly, molecules with both fluorescence and LC characteristics are promising candidates for developing functional materials such as fluorescence sensors and fluorescence thermometers, which can reversibly alter their fluorescence behavior through a temperature- and concentration-dependent control of their aggregated structures [9,10].

**Figure 1:**
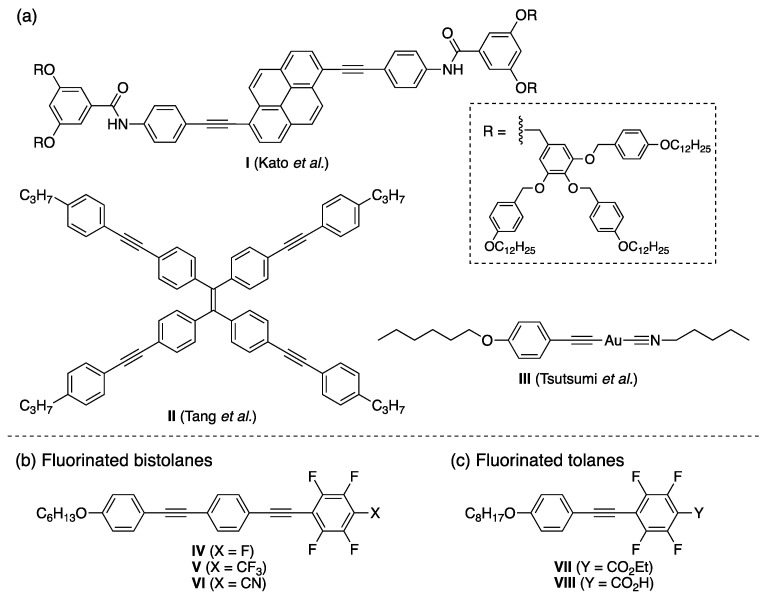
Chemical structures of (**a**) PLLC molecules **I**–**III** obtained from the literature [11,12,13] and (**b**) fluorinated bistolane-type PLLCs **IV–VI** [14,15] and (**c**) fluorinated tolane-type PLLCs **VII** and **VIII** developed by our group [16,17].

In 2008, Kato et al. reported that the color of photoluminescence of pyrene derivatives **I** with dendritic flexible moieties changes from yellow to blue-green owing to their phase transition from the cubic phase to the shear-induced columnar phase [11]. Since then, various photoluminescent (PL) LC molecules, such as **II** and **III,** have been developed and investigated in detail by Tang et al. [12] and Tsutsumi et al. [13], respectively (Figure 1a).

Our research group has successfully developed a family of donor-π-acceptor (D-π-A)-type fluorinated bistolane derivatives **IV** to **VI**; however, their synthetic procedures were tedious and inefficient (Figure 1b) [14,15]. Furthermore, our group has also developed PLLC molecules **VII** and **VIII** (Figure 1c) using fluorinated tolanes as mesogens, which are easy to synthesize [16,17]. These molecules exhibit a nematic (N) phase with the LC phase as the only orientational order, and their Cry–N phase transition significantly reduces the fluorescence quantum yields (Φ_PL_). Therefore, the combination of higher-order LC phases, such as smectic (Sm) phases, with the PL properties of the condensed phase can produce high photoluminescence even in the LC phase.

**Figure 2:**
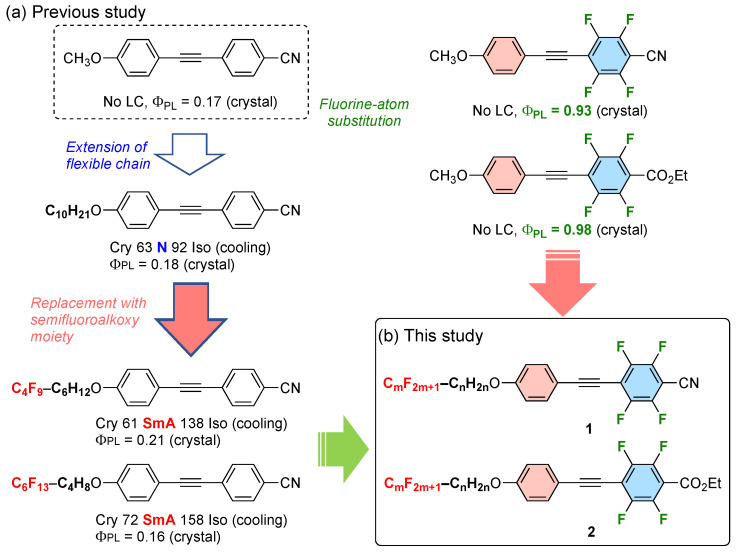
(**a**) Chemical structures and representative properties of molecules reported in our previous study and (**b**) chemical structures of semifluoroalkoxy-containing D-π-A-type fluorinated tolanes **1** and **2** developed in the present work.

It has been shown that incorporating a semifluoroalkyl flexible chain unit into a mesogenic core is an effective molecular design approach to effectively express the Sm LC phase [18,19]. In fact, our recent study has reported that incorporating a semifluoroalkoxy fragment into D-π-A-type nonfluorinated tolane exhibits a smectic A (SmA) phase as well as weak photoluminescence (Figure 2a) [20]. Based on the knowledge of fluorinated PL molecules in our group, we conceived that a D-π-A-type fluorinated diphenylacetylene tolane containing a semifluoroalkoxy chain provides efficient PLLC molecules that exhibit efficient PL in the condensed phase (Figure 2b). In this study, we investigate the photophysical and phase transition behavior of two semifluoroalkoxy-containing D-π-A-type fluorinated tolanes, (1) **1** with a 2,3,5,6-tetrafluorobenzonitrile moiety and (2) **2** with a 2,3,5,6-tetrafluorobenzoate partial structure.

## 2. Results and Discussion

### 2.1. Synthesis

**Scheme 1:**
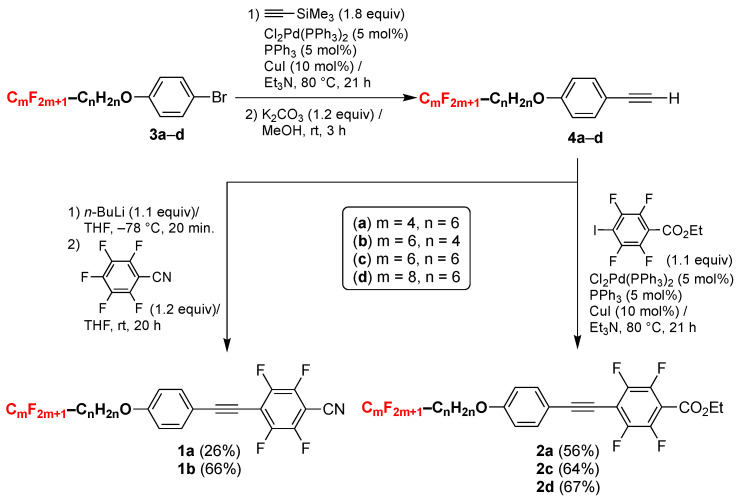
Synthesis scheme of semifluoroalkoxy-containing D-π-A-type fluorinated diphenylacetylenes **1** and **2**.

Syntheses of semifluoroalkoxy-containing D-π-A type fluorinated tolanes **1** and **2** were investigated according to the scheme shown in Scheme 1.

Syntheses of CN-terminated **1** and CO_2_Et-terminated **2** were performed using the common synthetic intermediate **4**, which was prepared by a Pd(0)-catalyzed Sonogashira cross-coupling reaction according to our previous report [16,20]. The CN-terminated molecules **1a** and **1b** were synthesized in 26% and 66% yield, respectively, via an addition–elimination reaction between pentafluorobenzonitrile and 4-semifluoroalkoxy-substituted phenylethynyllithium, which was readily prepared from **4**. The CO_2_Et-terminated molecules **2a**, **2c**, and **2d** were obtained in 56–67% yield through a Pd(0)-catalyzed Sonogashira cross-coupling reaction between **4** and ethyl 2,3,5,6-tetrafluoro-4-iodobenzoate. The resulting fluorinated tolanes were purified by silica gel column chromatography followed by recrystallization. Nuclear magnetic resonance (NMR) spectroscopic measurements showed that the purities of the resulting tolanes were suitable for evaluating their photophysical and phase transition behavior.

### 2.2. Photophysical Behavior

**Figure 3:**
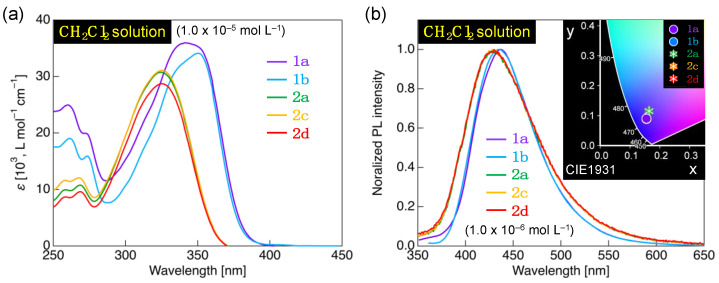
(**a**) UV–vis absorption (concentration: 1.0 × 10^−5^ mol L^−1^) and (**b**) PL spectra (concentration: 1.0 × 10^−6^ mol L^−1^) of semifluoroalkoxy-containing D-π-A-type fluorinated tolanes **1** and **2**. Inset: Commission Internationale de l’Eclailage plot of their PL colors.

With the semifluoroalkoxy-containing D-π-A-type fluorinated tolanes **1** and **2** in hand, we initially evaluated the solution-phase photophysical behavior of the semifluoroalkoxy-containing D-π-A-type fluorinated tolanes **1** and **2**. The solution sample was prepared by dissolving the crystalline sample with dichloromethane, and its concentration was adjusted to 1.0 × 10^−5^ mol L^−1^ for ultraviolet–visible (UV–vis) light absorption measurements and 1.0 × 10^−6^ mol L^−1^ for photoluminescence measurements. Figure 3 shows the UV–vis absorption and PL spectra of **1** and **2** in a CH_2_Cl_2_ solution and the Commission Internationale de l’Eclailage (CIE) plot to quantitatively assess their PL colors. Photophysical data obtained are summarized in Table 1.

Both CN-terminated D-π-A-type fluorinated tolanes (**1a** and **1b**) and CO_2_Et-terminated analogues (**2a**, **2c**, and **2d**) exhibited two main absorption bands, that is, short- and long-wavelength bands below and above 300 nm of the maximum absorption wavelength (*λ*_abs_), respectively. The absorption bands in the short wavelength region were almost the same, regardless of the terminal substituents to which the fluorinated aromatic rings were attached. In contrast, the *λ*_abs_ of **1a** and **1b** with a CN group shifted to the longer wavelength region compared to those of **2a**, **2c**, and **2d** with a CO_2_Et group owing to the changes in the energy gaps between the highest occupied molecular orbital (HOMO) and the lowest unoccupied molecular orbital (LUMO) of **1** with a CN group and **2** with a CO_2_Et group (Figures S22–S25). To understand vertical electronic transitions in **1** and **2**, theoretical evaluation of **1a** and **2a** was conducted using time-dependent density functional theory (TD-DFT) calculations with the M06-2X/6-31+G(d,p) level of theory. As shown in Figures S22 and S24, the two following allowed transitions were calculated: HOMO–2 → LUMO and HOMO → LUMO in **1a**, and HOMO–1 → LUMO and HOMO → LUMO in **2a**. HOMO–2 in **1a** and HOMO–1 in **2a** are the molecular orbitals localized on the fluorinated aromatic rings, while HOMO and LUMO are the orbitals covering the entire π-conjugated core. Considering the molecular orbital distributions of **1a** and **2a**, the short- and long-wavelength absorption bands originate from the ππ* transitions involving local excitation and intermolecular charge transfer, respectively.

Next, when a 1.0 × 10^−6^ mol L^−1^ CH_2_Cl_2_ solution of **1a** was irradiated with long-wavelength light out of the *λ*_abs_, a single PL band around the PL maximum wavelength (*λ*_PL_) of 437 nm was observed emitting blue fluorescence at (*x*, *y*) = (0.155, 0.090) CIE coordinates. The *λ*_PL_ of **1b** with a CN group was nearly identical to that of **1a**, while **2a** with the CO_2_Et group exhibited a slight blue-shift (428 nm) of the *λ*_PL_, in which the CIE coordinates were (*x*, *y*) = (0.164, 0.115). The blue-shift of the *λ*_PL_ is also attributed to the widening of the HOMO–LUMO energy gap with change from the CN group to the CO_2_Et group. It was found that the photophysical behavior of the D-π-A-type fluorinated tolanes significantly affects only the electron-density distribution on the π-conjugated framework and not that on the semifluoroalkoxy flexible unit. The Φ_PL_ of the CN-terminated molecules **1a** and **1b** was observed to be up to 0.20, which was due to quick internal conversion from the linearly shaped radiative ππ* excited state to the *trans*-bend-shaped non-radiative πσ* excited state [21,22]. On the other hand, a slightly better Φ_PL_ (up to 0.42) was observed for the CO_2_Et-terminated analogues compared to that of the CN-terminated counterparts, with suppressed fluorescence self-quenching due to the overlap of absorption and PL spectra [23].

**Figure 4:**
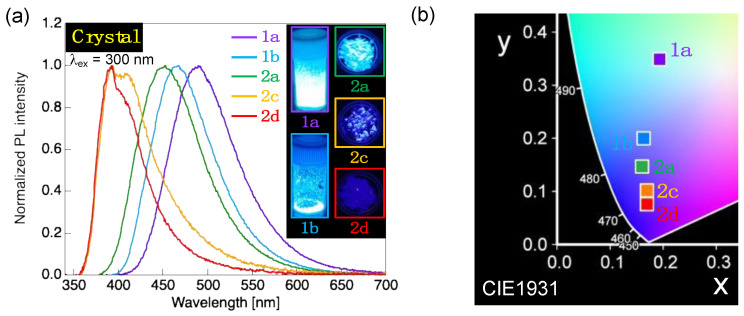
(**a**) PL spectra of semifluoroalkoxy-containing D-π-A-type fluorinated tolanes **1** and **2** in the crystalline phase (*λ*_ex_ = 300 nm). Inset: Photographs of PL by the tolanes under UV irradiation (*λ*_ex_ = 365 nm). (**b**) CIE plot of the tolanes calculated from their PL spectra.

Moreover, CN-terminated **1** and CO_2_Et-terminated **2** exhibited PL even in the crystalline (Cry) phase. The PL spectra and CIE plot are shown in Figure 4, and the obtained photophysical data are summarized in Table 1.

Thus, **1a** had a single PL band with a *λ*_PL_ around 490 nm and a light-blue PL at (*x*, *y*) = (0.193, 0.349) CIE coordinates with a high PL efficiency (Φ_PL_ = 0.61). Altering flexible unit from a nonafluorodecyloxy of **1a** to a tridecafluorodecyloxy chain of **1b** formed loose aggregates due to higher fluorine contents, leading to blue PL at (*x*, *y*) = (0.163, 0.200) CIE coordinates with a blue-shifted *λ*_PL_ band around 467 nm and a high Φ_PL_ of 0.71. Considering similar PL spectra in solution, the change in *λ*_PL_ between **1a** and **1b** seems to be caused by differences in aggregated structure in the crystalline lattice, supported by PXRD measurements at 25 °C (Figure S21). The Φ_PL_s of the CN-terminated molecules **1a** and **1b** in Cry phase were significantly higher than those of the dilute solution of **1a** and **1b** because of the significantly suppressed non-radiative deactivation due to the formation of intermolecular H···F hydrogen bonds. The CO_2_Et-terminated molecule **2a** exhibited blue PL at (*x*, *y*) = (0.160, 0.147) CIE coordinates with a single PL band around 453 nm of *λ*_PL_ with a relatively high Φ_PL_ (0.48). On the other hand, **2c** and **2d** exhibited two PL bands around 392–394 nm and 408–409 nm of *λ*_PL_ to show deep blue PL at (*x*, *y*) = (0.169, 0.102) and (*x*, *y*) = (0.169, 0.076) CIE coordinates, respectively, with low PL efficiency. According to the results of PXRD measurements at 25 °C in **2a**, **2c**, and **2d** (Figure S21), the three molecules showed different diffraction peaks, which suggests that they formed different aggregated structures in the crystalline phase through a variety of intermolecular interactions. It is considered that **2a**, **2c**, and **2d** showed different PL behavior due to different aggregate structures resulting from their various intermolecular interactions. Fluorinated tolanes, such as **2a**, **2c**, and **2d**, have a sterically bulky CO_2_Et group compared to the CN units of **1a** and **1b**, leading to a significant decrease in their Φ_PL_ due to the formation of loose aggregates that facilitate non-radioactive deactivation through molecular motion.

### 2.3. Phase Transition Behavior

We also focused on the phase transition behavior of the semifluoroalkoxy-containing D-π-A-type fluorinated tolanes **1** and **2** with condensed-phase PL characteristics. Phase transition behavior of **1** and **2** was evaluated with differential scanning calorimetry (DSC) and polarized optical microscopy (POM). The phase transition sequences and temperatures of **1a**, **1b**, **2a**, **2c**, and **2d** are summarized in Table 2.

**Figure 5:**
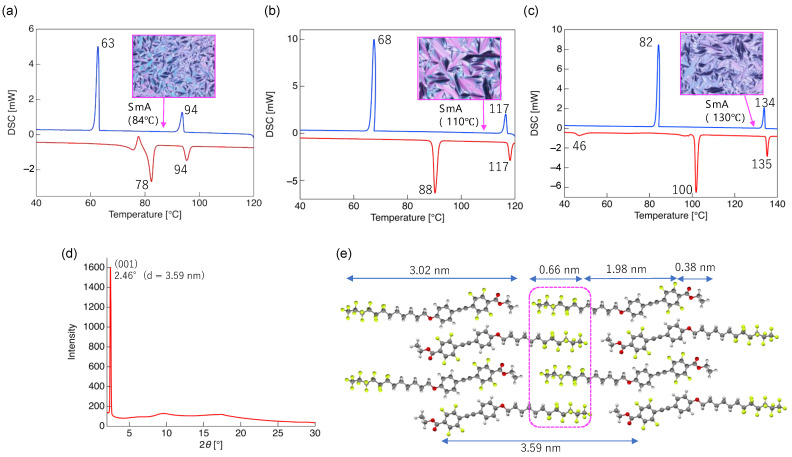
DSC thermogram and POM optical texture in the mesophases of (**a**) **2a**, (**b**) **2c**, and (**c**) **2d**. Scan rate: 5.0 °C min^−1^. (**d**) VT-PXRD pattern and (**e**) possible aggregated structures of **2c** in the mesophase.

Although semifluoroalkoxy-containing D-π-A-type nonfluorinated tolanes were reported to exhibit an SmA LC phase [20], the LC phase disappeared in the case of fluorinated tolane scaffolds. The phase transition enthalpies between the Cry and isotropic (Iso) phases of **1a** and **1b** were very large, which is attributed to the remarkable stabilization of the Cry phase by the introduction of fluorine atoms on the tolane backbone. In contrast to CN-terminated **1a** and **1b**, the CO_2_Et-terminated **2** exhibited a mesophase between the Cry and Iso phases (Figure 5).

During the second heating process, **2a** with a nonafluorodecyloxy chain (F content in the flexible chain: 54%) transitioned from Cry to mesophase at 80 °C and to Iso phase at 94 °C. The POM measurements revealed that the focal conic fan texture was in the mesophase; therefore, the observed mesophase was determined to be the SmA phase. Furthermore, **2c** with a tridecafluorododecyloxy chain (F content in the flexible chain: 59%) also exhibited an SmA phase with a focal conic fan texture at a temperature range of 89–117 °C during the second heating process. Increasing the fluorine atom contents within the flexible chain structure broadened the LC temperature range. During the heating and cooling processes, **2d** with a heptadecafluorotetradecyloxy chain (F content in the flexible chain: 62%) also exhibited an SmA phase, and the LC phase was observed to be in a range of 101–135 °C, which was wider than the others. To reliably identify the SmA phases observed in **2a**, **2c**, and **2d**, temperature-variable powder X-ray diffraction (VT-PXRD) measurements were performed at the mesophase temperature. In **2a**, which has a short fluoroalkyl moiety, no diffraction peak corresponding to the (001) plane index was observed (Figure S18a). It is considered that the fluorine atoms in the flexible chain formed an interdigitate structures, which formed many defects in the layer structure and reduced the domain size [24,25]. In contrast, **2b** and **2c**, which has an elongated fluoroalkyl structure, exhibited diffraction peaks corresponding to the (001) plane at 2*θ* = 2.46° and 2.22°, respectively (Figure 5d and Figure S18e). Using Bragg’s equation, the interlayer distances (*d*-spacing) in **2c** and **2d** were calculated as 3.59 nm and 3.97 nm, respectively. Considering the molecular lengths of **2c** (3.02 nm) and **2d** (3.28 nm) estimated by quantum chemical calculation (Figure 5e and Figure S26), it is anticipated that the structure in which the fluoroalkyl moieties are interdigitated and the π-conjugated mesogen is intercalated is the aggregated structure in the SmA phase (Figure 5e).

### 2.4. PL Behavior in the SmA Phase

**Figure 6:**
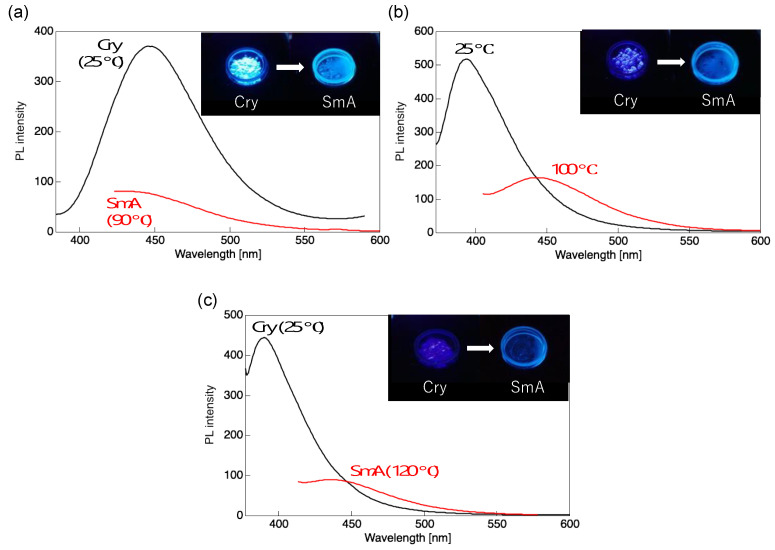
PL spectra of (**a**) **2a**, (**b**) **2c**, and (**c**) **2d** in Cry and SmA phases. Excitation wavelength: 380 nm for **2a**, 362 nm for **2c**, and 392 nm for **2d**.

We evaluated the photophysical behavior of **2a**, **2c**, and **2d**, which have both crystalline-state PL and LC properties, in the SmA phase (Figure 6, Table 3). Using a fluorescence spectrophotometer equipped with a hand-made heating unit containing a ceramic heater and a temperature control device, PL spectra of **2a**, **2c**, **2d** were measured under thermal conditions.

Focusing on the *λ*_PL_ in the SmA phase under thermal conditions, as shown in Figure 6 and Table 3, a blue shift of approximately 20 nm was observed for **2a**. In contrast, the *λ*_PL_ bands of **2c** and **2d** red-shifted by up to 51 nm during the Cry–SmA phase transition, which is due to the formation of π–π stacking induced by interdigitation of fluoroalkoxy moieties. Furthermore, the fluorescence intensity of the SmA phase under heating was significantly decreased compared to that of the Cry phase at 25 °C. The ratio of PL intensities in the SmA and Cry phases, *I*_SmA_/*I*_Cry_, was 0.22 for **2a**, 0.32 for **2c**, and 0.20 for **2d**. This is due to acceleration of non-radiative deactivation caused by the micro-Brownian motion in the appearance of the SmA phase.

## 3. Materials and Methods

### 3.1. General

Semifluoroalkoxy-substituted fluorinated tolanes, **1** and **2**, were synthesized according to the scheme shown in Figure 3. The reaction progress was confirmed using thin-layer chromatography (TLC), which was performed on silica gel TLC plates (silica gel 60254, Merck, Rahway, NJ, USA). The tolanes were purified by column chromatography using Wakogel^®^ 60N (38–100 mm). Melting and clearing temperatures of the molecules **1** and **2** were determined using DSC. ^1^H and ^13^C-NMR spectra for **1** and **2** were recorded using a Bruker AVANCE III 400 NMR spectrometer (^1^H: 400 MHz and ^13^C: 100 MHz) in chloroform-*d* (CDCl_3_), and the chemical shifts were reported in parts per million (ppm) using the residual proton in the NMR solvent. ^19^F-NMR (376 MHz) spectra were obtained using a Bruker AVANCE III 400 NMR spectrometer in CDCl_3_, and trichlorofluoromethane (CFCl_3_, *d*_F_ = 0 ppm) or hexafluorobenzene (C_6_F_6_, *d*_F_ = −163 ppm) was used as an internal standard. Infrared (IR) spectra were recorded using the KBr method with a JASCO FT/IR-4100 type A spectrometer, and all spectra were reported in wavenumber (cm^−1^). High-resolution mass spectrometry (HRMS) was performed on a JEOL JMS-700MS spectrometer using the fast atom bombardment (FAB) method.

### 3.2. Synthesis Procedure of 2,3,5,6-Tetrafluoro-4-[2-{4-(7,7,8,8,9,9,10,10,10-nonafluorodecyloxy)phenyl}ethyn-1-yl]benzonitrile (***1a***)

In a two-necked round bottomed flask equipped with a Teflon^®^-coated stirring bar, 4-(7,7,8,8,9,9,10,10,10-nonafluorodecyloxy)phenylacetylene **4a** (0.824 g, 2.0 mmol) and tetrahydrofuran (THF, 20 mL) were mixed. n-BuLi was added dropwise to the solution at −78 °C, and the mixture was continuously stirred at −78 °C for 20 min. Then, 2,3,4,5,6-pentafluorobenzonitrile (0.454 g, 2.4 mmol) was added to the solution, and the reaction temperature was increased to room temperature (25 °C). After this, the solution was stirred at room temperature for 20 h. After stirring for 20 h, the precipitate was separated by atmospheric filtration, and the filtrate was poured into an aqueous NH4Cl solution (10 mL). The crude product was extracted using EtOAc (10 mL, three times), and the combined organic layer was washed with brine (once). Furthermore, the collected organic layer was dried over anhydrous Na_2_SO_4_, which was separated by filtration. The filtrate was evaporated in vacuo and subjected to silicagel column chromatography (eluent: hexane/EtOAc = 30/1), followed by recrystallization using CHCl_3_/hexane (*v*/*v* = 2/1), to obtain **1a** in 26% yield (0.320 g, 0.5 mmol) as a white solid.

#### 3.2.1. 2,3,5,6-Tetrafluoro-4-[2-{4-(7,7,8,8,9,9,10,10,10-nonafluorodecyloxy)phenyl}ethyn-1-yl]benzonitrile (**1a**)

Yield: 26% (white solid), m.p. = 110 °C determined by DSC; ^1^H-NMR (CDCl_3_): *δ* 1.44–1.60 (m, 4H), 1.66 (quin, *J* = 7.2 Hz, 2H), 1.83 (quin, *J* = 7.6 Hz, 2H), 2.01–2.16 (m, 2H), 4.01 (t, *J* = 6.4 Hz, 2H), 6.91 (d, *J =* 8.8 Hz, 2H), 7.54 (d, *J =* 8.8 Hz, 2H); ^13^C-NMR (CDCl_3_): *δ* 20.1, 25.7, 28.81, 28.85, 30.7 (t, *J* = 22.0 Hz), 67.9, 72.9, 92.9 (d, *J* = 19.8 Hz), 107.5 (dd, *J* = 6.6, 3.7 Hz), 107.7 (t, *J* = 3.7 Hz), 109–112 (m, 1C for quaternary carbon of fluorinated benzene), 105–120 (m, 4C for C_4_F_9_ moiety), 112.5, 114.8, 134.0, 146.3 (dd, *J* = 255.9, 16.8 Hz), 147.0 (dd, *J* = 256.0, 14.6 Hz), 160.9; ^19^F-NMR (CDCl_3_, CFCl_3_): *δ* −82.33 (t, *J =* 9.8 Hz, 3F), −115.0 to −115.28 (m, 2F), −124.92 to −125.13 (m, 2F), −126.48 to −126.56 (m, 2F), −133.63 to −133.79 (m, 2F), −134.57 to −134.73 (m, 2F); IR (KBr) *ν* 2952, 2861, 2262, 2216, 1646, 1519, 1471, 1332, 1294, 1259, 1201, 1075, 835 cm^−1^; HRMS (FAB) Calcd for (M+) C25H16F13NO: 593.1024, observed: 593.1024.

#### 3.2.2. 2,3,5,6-Tetrafluoro-4-[2-{4-(5,5,6,6,7,7,8,8,9,9,10,10,10-tridecafluorodecyloxy)phenyl}ethyn-1-yl]benzonitrile (**1b**)

Yield: 56% (white solid), m.p. = 71 °C determined by DSC; ^1^H-NMR (CDCl_3_): *δ* 1.79–1.97 (m, 4H), 2.09–2.26 (m, 2H), 4.05 (t, *J* = 6.0 Hz, 2H), 6.92 (d, *J =* 8.8 Hz, 2H), 7.55 (d, *J =* 8.8 Hz, 2H); ^13^C-NMR (CDCl_3_): *δ* 17.3, 28.6, 30.7 (t, *J* = 22.7 Hz), 67.4, 73.0 (t, *J* = 4.4 Hz), 93.0 (d, *J* = 17.6 Hz), 107.5 (d, *J* = 3.7 Hz, two carbons are overlapped), 109–112 (m, 1C for quaternary carbon of fluorinated benzene), 105–120 (m, 6C for C_6_F_13_ moiety), 112.8, 114.8, 134.1, 146.3 (dd, *J* = 254.5, 11.8 Hz), 147.0 (dd, *J* = 260.4, 15.4 Hz), 160.6; ^19^F-NMR (CDCl_3_, C_6_F_6_): *δ* −82.03 (t, *J =* 9.8 Hz, 3F), −115.66 (quin, *J* = 17.7 Hz, 2F), −123.0 to −123.3 (m, 2F), −124.0 to −124.3 (m, 2F), −124.6 to −124.9 (m, 2F), −127.2 to −127.5 (m, 2F), −134.32 to −134.45 (m, 2F), −135.28 to −135.42 (m, 2F); IR (KBr) *ν* 2965, 2888, 2217, 1646, 1562, 1519, 1463, 1365, 1332, 1293, 1123, 1076, 985 cm^−1^; HRMS (FAB) Calcd for (M+) C25H12F17NO: 665.0647, observed: 665.0655.

### 3.3. Synthesis Procedure of Ethyl 2,3,5,6-Tetrafluoro-4-[2-{4-(7,7,8,8,9,9,10,10,10-nonafluorodecyloxy)phenyl}Ethyn-1-yl]benzoate (***2a***)

In a two-necked round-bottomed flask equipped with a Teflon^®^-coated stirring bar, 4-(7,7,8,8,9,9,10,10,10-nonafluorodecyloxy)phenylacetylene **4a** (1.050 g, 2.5 mmol), Cl2(PPh3)2Pd(II) (0.087 g, 0.12 mmol), ethyl 2,3,5,6-tetrafluoro-4-iodobenzoate (1.130 g, 3.3 mmol), and PPh3 (0.033 g, 0.12 mmol) in Et3N (20 mL) were placed. Then, CuI(I) (0.048 g, 0.25 mmol) was added to the suspended solution. The mixture was heated at 80 °C and stirred for 21 h. Then, the precipitate was separated through atmospheric filtration, and the filtrate was poured into an aqueous NH4Cl solution (10 mL). The crude product was extracted with EtOAc (10 mL, three times), and the combined organic layer was washed with brine (once). After this, the collected organic layer was dried over anhydrous Na_2_SO_4_, which was separated by filtration. The filtrate was evaporated in vacuo and subjected to silica-gel column chromatography (eluent: hexane/EtOAc = 30/1), followed by recrystallization from CHCl_3_/MeOH (*v*/*v* = 2/1) to obtain the title product **2a** in 56% yield (0.900 g, 1.4 mmol) as a white solid.

#### 3.3.1. Ethyl 2,3,5,6-Tetrafluoro-4-[2-{4-(7,7,8,8,9,9,10,10,10-nonafluorodecyloxy)phenyl}ethyn-1-yl]benzoate (**2a**)

Yield: 56% (white solid), m.p. = 71 °C determined by DSC; ^1^H-NMR (CDCl_3_): *δ* 1.41 (t, *J =* 6.8 Hz, 3H), 1.46–1.53 (m, 4H), 1.66 (quin, *J* = 7.6 Hz, 2H), 1.83 (quin, *J* = 7.6 Hz, 2H), 2.00–2.16 (m, 2H), 4.00 (t, *J* = 6.4 Hz, 2H), 4.45 (q, *J =* 7.2 Hz, 2H), 6.90 (d, *J =* 8.8 Hz, 2H), 7.53 (d, *J =* 8.8 Hz, 2H); ^13^C-NMR (CDCl_3_): *δ* 13.9, 20.0 (t, *J* = 2.9 Hz), 25.7, 28.8, 28.9, 30.7 (t, *J =* 22.7 Hz), 62.7, 67.8, 72.9 (t, *J* = 3.7 Hz), 104.4 (t, *J =* 3.6 Hz), 105–120 (m, 4C for C_4_F_9_ moiety), 107.9 (t, *J* = 17.6 Hz), 112.0 (t, *J =* 16.1 Hz), 113.1, 114.6, 133.7, 144.5 (ddt, *J* = 255.2, 14.7, 5.1 Hz), 146.5 (ddt, *J* = 253.8, 13.9, 3.7 Hz), 159.3, 160.5; ^19^F-NMR (CDCl_3_, C_6_F_6_): *δ* −82.3 (t, *J =* 9.9 Hz, 3F), −115.82 to −116.03 (m, 2F), −125.70 to −125.88 (m, 2F), −127.28 to −127.46 (m, 2F), −137.44 to −137.59 (m, 2F), −141.45 to −141.62 (m, 2F); IR (KBr) *ν* 2948, 2223, 1730, 1476, 1334, 1205, 1132, 984, 837 cm^−1^; HRMS (FAB) Calcd for (M+) C27H21F13O3: 640.1283, observed: 640.1275.

#### 3.3.2. Ethyl 2,3,5,6-Tetrafluoro-4-[2-{4-(7,7,8,8,9,9,10,10,11,11,12,12,12-trideccafluorododecyloxy)phenyl}ethyn-1-yl]benzoate (**2c**)

Yield: 64% (white solid), m.p. = 89 °C determined by DSC; ^1^H-NMR (CDCl_3_): *δ* 1.41 (t, *J =* 6.8 Hz, 3H), 1.44–1.57 (m, 4H), 1.65 (quin, *J* = 7.6 Hz, 2H), 1.82 (quin, *J =* 7.6 Hz, 2H), 1.99–2.16 (m, 2H), 3.98 (t, *J* = 6.4 Hz, 2H), 4.45 (q, *J =* 6.8 Hz, 2H), 6.88 (d, *J =* 8.8 Hz, 2H), 7.51 (d, *J =* 8.8 Hz, 2H); ^13^C-NMR (CDCl_3_): *δ* 13.9, 20.1 (t, *J* = 3.6 Hz), 25.7, 28.8, 28.9, 30.8 (t, *J =* 22.0 Hz), 62.7, 67.8, 73.0 (t, *J* = 4.4 Hz), 104.4 (t, J = 3.7 Hz), 105–120 (m, 6C for C_6_F_13_ moiety), 108.0 (t, *J* = 17.6 Hz), 112.1 (t, *J* = 16.2 Hz), 113.2, 114.7, 133.7, 144.6 (ddt, *J* = 255.9, 14.0, 5.1 Hz), 146.5 (ddt, *J* = 253.8, 14.6, 3.0 Hz), 159.5, 160.4; ^19^F-NMR (CDCl_3_,C_6_F_6_): *δ* −81.90 (t, *J =* 9.9 Hz, 3F), −115.32 to −115.58 (m, 2F), −122.78 to −123.07 (m, 2F), −123.74 to −124.03 (m, 2F), −124.44 to −124.67 (m, 2F), −127.08 to −127.26 (m, 2F), −137.28 to −137.44 (m, 2F), −141.22 to −141.39 (m, 2F); IR (KBr) *ν* 2947, 2216, 1738, 1475, 1331, 1201, 1141, 987 cm^−1^; HRMS (FAB) Calcd for (M+) C29H21F17O3: 740.1219, observed: 740.1223.

#### 3.3.3. Ethyl 2,3,5,6-Tetrafluoro-4-[2-{4-(7,7,8,8,9,9,10,10,11,11,12,12,13,13,14,14,14-heptadeccafluorotetradecyloxy)phenyl}ethyn-1-yl]benzoate (**2d**)

Yield: 67% (white solid), m.p. = 108 °C determined by DSC; ^1^H-NMR (CDCl_3_): *δ* 1.41 (t, *J =* 6.8 Hz, 3H), 1.44–1.58 (m, 4H), 1.65 (quin, *J* = 7.6 Hz, 2H), 1.82 (quin, *J =* 7.6 Hz, 2H), 1.98–2.15 (m, 2H), 3.98 (t, *J =* 6.4 Hz, 2H), 4.45 (q, *J =* 7.2 Hz, 2H), 6.88 (d, *J =* 8.8 Hz, 2H), 7.52 (d, *J =* 8.8 Hz, 2H); ^13^C-NMR (CDCl_3_): *δ* 14.0, 20.1 (t, *J* = 2.9 Hz), 25.7, 28.8, 28.9, 30.8 (t, *J* = 22.0 Hz), 62.7, 67.8, 73.0 (t, *J* = 3.7 Hz), 104.4 (t, *J* = 3.7 Hz), 105–120 (m, 8C for C_8_F_17_ moiety), 108.0 (t, *J* = 17.6 Hz), 112.1 (t, *J* = 16.2 Hz), 113.2, 114.6, 133.7, 144.5 (ddt, *J* = 256.8, 14.7, 5.1 Hz), 146.5 (ddt, *J* = 253.8, 14.7, 4.4 Hz), 159.5, 160.4; ^19^F-NMR (CDCl_3_, CFCl3): *δ* −81.05 (t, J = 9.4 Hz, 3F), −114.42 to −114.76 (m, 2F), −121.9 (brs, 2F), −122.1 (brs, 2F), −123.7 (brs, 2F), −126.3 (brs, 2F), −136.35 to −136.58 (m, 2F), −140.34 to −140.54 (m, 2F); IR (KBr) *ν* 2947, 2216, 1738, 1475, 1331, 1202, 1147, 833 cm^−1^; HRMS (FAB) Calcd for (M+) C31H21F21O3: 840.1155, observed: 840.1162.

### 3.4. Theoretical Assessment

All DFT calculations were performed using the Gaussian 16 program set [26] with the M06-2X hybrid functional [27] and 6-31+G(d,p) (for all atoms) basis set. A conductor-like polarizable continuum model (CPCM) [28] was used for CH_2_Cl_2_. Theoretical vertical transitions were also calculated by TD-DFT at the same theory level using the same solvation model.

### 3.5. Photophysical Behavior

UV–vis absorption spectra were recorded using a JASCO V-750 absorption spectrometer (JASCO, Tokyo, Japan). The PL spectra of the solutions were measured using an FP-6600 fluorescence spectrometer (JASCO, Tokyo, Japan). The PL quantum yields were measured using a Quantaurus-QY C11347-01 instrument (Hamamatsu Photonics, Hamamatsu, Japan). The PL lifetime was measured using a Quantaurus-Tau fluorescence lifetime spectrometer (C11367-34, Hamamatsu Photonics, Japan).

### 3.6. Phase Transition Behavior

The phase transition behaviors were observed by POM using an Olympus BX53 mi-croscope (Tokyo, Japan) equipped with a cooling and heating stage (10002L, Linkam Sci-entific Instruments, Surrey, UK). Thermodynamic characterization was performed using DSC (DSC-60 Plus, Shimadzu, Kyoto, Japan) at heating and cooling rates of 5.0 °C min^−1^ under an N_2_ atmosphere. Liquid crystalline structures were evaluated by an FR-E X-ray diffractometer attached with an R-axis IV two-dimensional (2D) detector (Rigaku, Tokyo, Japan). 0.3 mm collimated Cu*K*a radiation (*λ* = 1.54187 Å) was used as an X-ray beam, and the camera length was set at 300 mm. The powder sample was loaded into a thin wall glass capillary tube for XRD analysis (*φ* 1.0–2.5 mm, Hilgenberg GmbH), and the annealed up to isotropic temperature under vacuum. The glass capillary was set onto a ceramic heater attached to the FR-E sample holder. Exposure time of the X-ray beam was 5 min.

## 4. Conclusions

We designed PLLCs, which exhibited both aggregation-state PL and SmA-type LC properties, and developed two types of semifluoroalkoxy-containing D-π-A-type fluorinated tolanes **1** with a tetrafluorobenzonitrile and **2** with a tetrafluorobenzoate moiety. CN-terminated **1** exhibited blue photoluminescence with a low PL quantum yield (Φ_PL_; up to 0.20) in a dilute solution phase; however, replacing the CN substituent with an CO_2_Et group increased the Φ_PL_ to up to 0.42. In contrast, CN-terminated **1** exhibited intense light-blue PL with a high Φ_PL_ of up to 0.71 in the crystalline phase, whereas CO_2_Et-terminated **2** exhibited blue PL with lower PL efficiency (Φ_PL_ = up to 0.48). In the phase transition characteristics, CN-terminated **1** did not exhibit any LC behavior upon the heating and cooling processes owing to the formation of tight molecular aggregates through H···F hydrogen bonds. Therefore, CO_2_Et-terminated **2** exhibited an enantiotropic SmA LC phase. Among **2a**, **2c**, and **2d**, the LC temperature range increased with increasing fluorine atom contents in the flexible chain. Using **2a**, **2c**, and **2d**, which have both SmA LC and aggregation state-PL characteristics, we investigated the PL behavior in the SmA phase. As a result, during the Cry–SmA phase transition, a significant decrease in fluorescence intensity was observed, whereas the PL color was altered during the phase transition. The molecular design and PLLC molecules discussed in this study provide novel temperature-dependent PL materials, which would become highly efficient thermal-stimulus responsive luminescent materials in the future.

## Figures and Tables

**Table 1:** Photophysical data of semifluoroalkoxy-containing D-π-A-type fluorinated tolanes **1** and **2** in CH_2_Cl_2_ solutions and crystalline states.

	CH_2_Cl_2_ Solution				Crystal	
Molecule	*λ*_abs_ [nm] ^1^ (*ε* [10^3^, L mol^–1^ cm^–1^])	*λ*_PL_ [nm] ^2^ (Φ_PL_) ^3^	CIE Coordinate (*x*, *y*)		*λ*_PL_ [nm] (Φ_PL_) ^3^	CIE Coordinate (*x*, *y*)
**1a**	260 (25.0), 272 (20.0), 341 (36.0)	437 (0.20)	(0.155, 0.090)		490 (0.61) ^4^	(0.193, 0.349)
**1b**	261 (19.0), 274 (15.9), 351 (34.1)	435 (0.18)	(0.155, 0.089)		467 (0.71) ^4^	(0.163, 0.200)
**2a**	259 (9.94), 269 (10.8), 324 (30.8)	428 (0.42)	(0.164, 0.115)		453 (0.48) ^4^	(0.160, 0.147)
**2c**	258 (11.6), 268 (12.0), 325 (31.1)	429 (0.42)	(0.163, 0.114)		394, 409 (0.13) ^5^	(0.169, 0.102)
**2d**	259 (8.68), 269 (9.61), 325 (28.7)	430 (0.39)	(0.164, 0.116)		392, 408sh (0.14) ^5^	(0.169, 0.076)

^1^ Concentration: 1.0 × 10^−5^ mol L^−1^. ^2^ Concentration: 1.0 × 10^−6^ mol L^−1^. ^3^ Measured using an integrated sphere. ^4^ Excitation wavelength: 310 nm. ^5^ Excitation wavelength: 350 nm.

**Table 2:** Phase transition sequences and temperatures during the second heating and cooling processes ^1^.

Molecule	Phase Transition Sequence and Temperature [°C] (Enthalpy [kJ mol^−1^])	
**1a**	Heating	Cry 109 (28.6) Iso
	Cooling	Cry 96 (−28.1) Iso
**1b**	Heating	Cry 113 (21.6) Iso
	Cooling	Cry 106 (−20.7) Iso
**2a**	Heating	Cry^1^ 77 (19.3) Cry^2^ 80 (22.4) SmA 94 (6.7) Iso
	Cooling	Cry^1^ 63 (−21.4) SmA 94 (−6.7) Iso
**2c**	Heating	Cry 89 (29.8) SmA 117 (8.2) Iso
	Cooling	Cry 67 (−27.7) SmA 117 (−8.3) Iso
**2d**	Heating	Cry^1^ 45 (5.3) Cry^2^ 101 (38.0) SmA 135 (10.1) Iso
	Cooling	Cry^1^ 39 (−5.4) Cry^2^ 84 (−35.1) SmA 134 (−10.1) Iso ^1^

^1^ Determined by DSC under nitrogen atmosphere (scan rate: 5 °C min^−1^).

**Table 3:** PL behavior difference between Cry and SmA phases in **2a**, **2c**, and **2d**.

Molecule	Phase	*λ*_PL_ [nm]	*I*_smA_/*I*_cry_
**2a**	Cry (25 °C)	448	0.22
	SmA (90 °C)	429	
**2c**	Cry (25 °C)	393	0.32
	SmA (100 °C)	444	
**2d**	Cry (25 °C)	390	0.20
	SmA (120 °C)	433	

## Data Availability

Not applicable.

## Supplementary Materials

The following supporting information can be downloaded at: https://www.mdpi.com/article/10.3390/molecules28062764/s1, Figures S1–S15: NMR spectra for ^1^H, ^13^C, and ^19^F nucleus; Figure S16: DSC thermograms; Figure S17: POM texture; Figure S18: VT-PXRD patterns; Figure S19: UV-vis absorption and PL spectra in CH_2_Cl_2_ solution; Figure S20: Excitation and PL spectra in Cry phase; Figure S21: PXRD patterns measured at 25 °C; Figure S22: Excitation and PL spectra in SmA phase; Figures S23–S26: Optimized structures and molecular orbital distributions; Figure S27: Possible aggregated structures of **2d** in the mesophase; Table S1: Phase transition behavior; Tables S2–S5: Cartesian coordinates.

## Sample Availability

Samples of the compounds are available from the authors.
