# Supplementary material for: Donor-π-Acceptor-Type Fluorinated Tolane Containing a Semifluoroalkoxy Chain as a Condensed-Phase Luminophore
Source: Molecules. 2023 Mar 19;28(6):2764. doi: 10.3390/molecules28062764 (PMC10055744; doi:10.3390/molecules28062764)
Supplement: Supplementary file 1 [file molecules-28-02764-s001.zip › molecules-2281740-supplementary.pdf]

*Supplementary Materials*

# Donor-π-Acceptor-Type Fluorinated Tolane Containing a Semifluoroalkoxy Chain as a Condensed-Phase Luminophore

**Shigeyuki Yamada [1,*,†], Keigo Yoshida [1,†], Mitsuki Kataoka [1,†], Mitsuo Hara [2] and Tsutomu Konno [1]**

[1] Faculty of Molecular Chemistry and Engineering, Kyoto Institute of Technology, Matsugasaki, Sakyo-ku, Kyoto 606-8585, Japan
[2] Department of Molecular and Macromolecular Chemistry, Graduate School of Engineering, Nagoya University, Furo-cho, Chikusa-ku, Nagoya, Aichi 464-8603, Japan
* Correspondence: syamada@kit.ac.jp; Tel.: +81-75-724-7517
† These authors contributed equally to this work.

## *Table of Contents*

NMR spectrum ••••••••••••••• S-2
Phase transition behavior ••••••••••••••• S-12
POM texture ••••••••••••••• S-13
VT-PXRD patterns ••••••••••••••• S-15
Photophysical behavior
  $CH_2Cl_2$ solution phase ••••••••••••••• S-16
  Cry phase ••••••••••••••• S-17
  SmA LC phase ••••••••••••••• S-19
Quantum chemical calculation ••••••••••••••• S-20
  Cartesian coordinate ••••••••••••••• S-25

## NMR spectrum

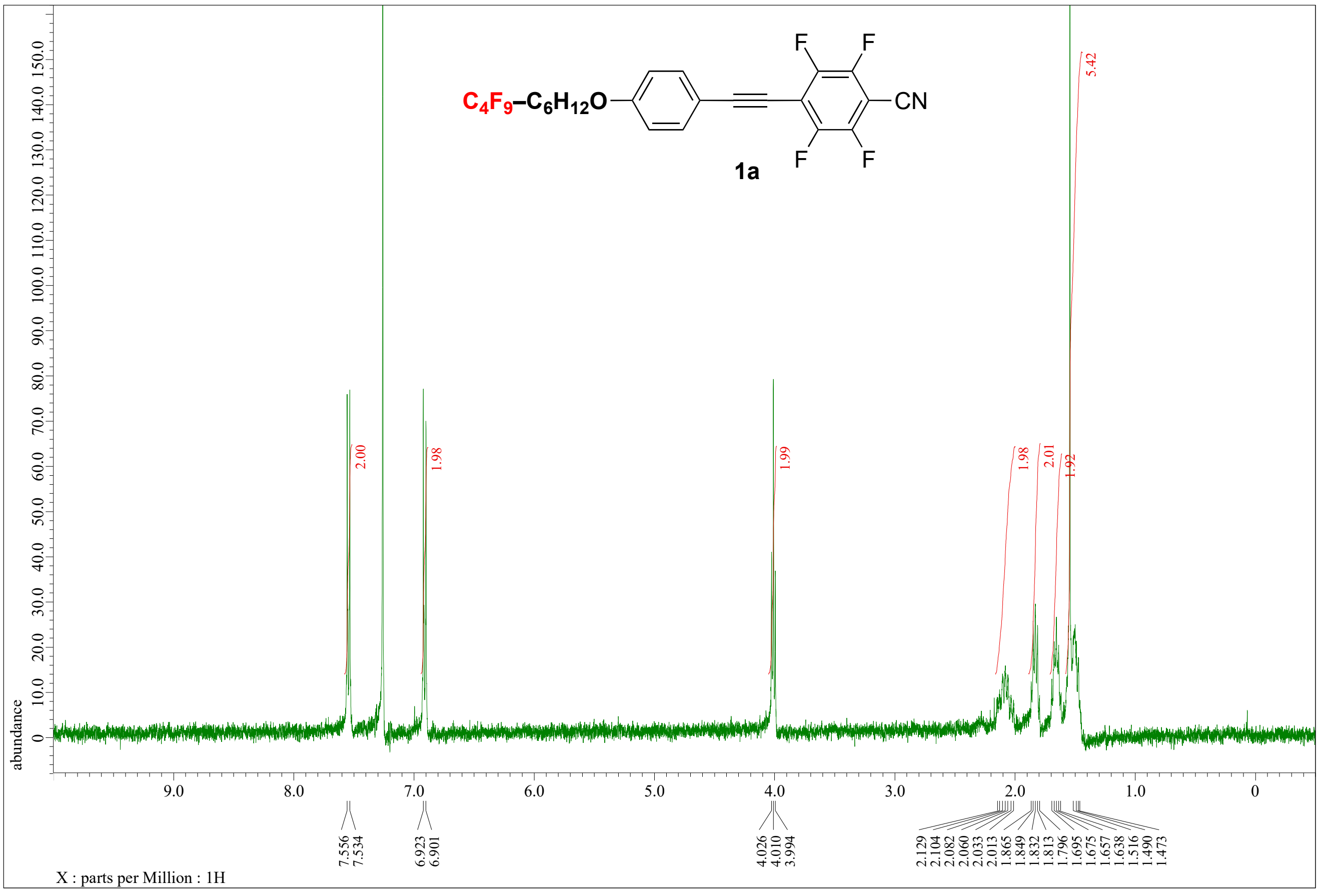


**Figure S1.** $^{1}$H NMR spectrum of **1a** ($CDCl_3$, 400 MHz).

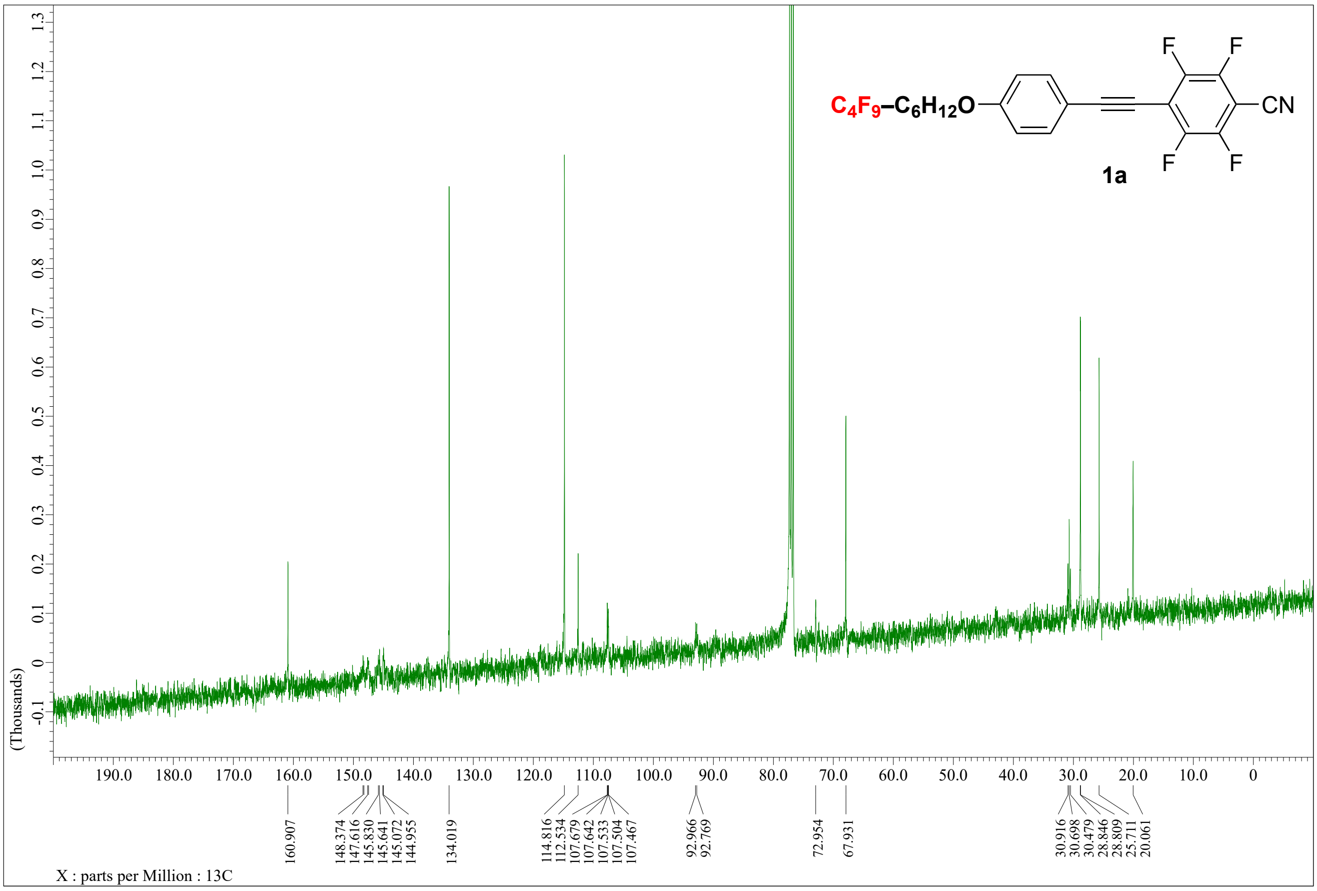


**Figure S2.** $^{13}$C NMR spectrum of **1a** ($CDCl_3$, 100 MHz).

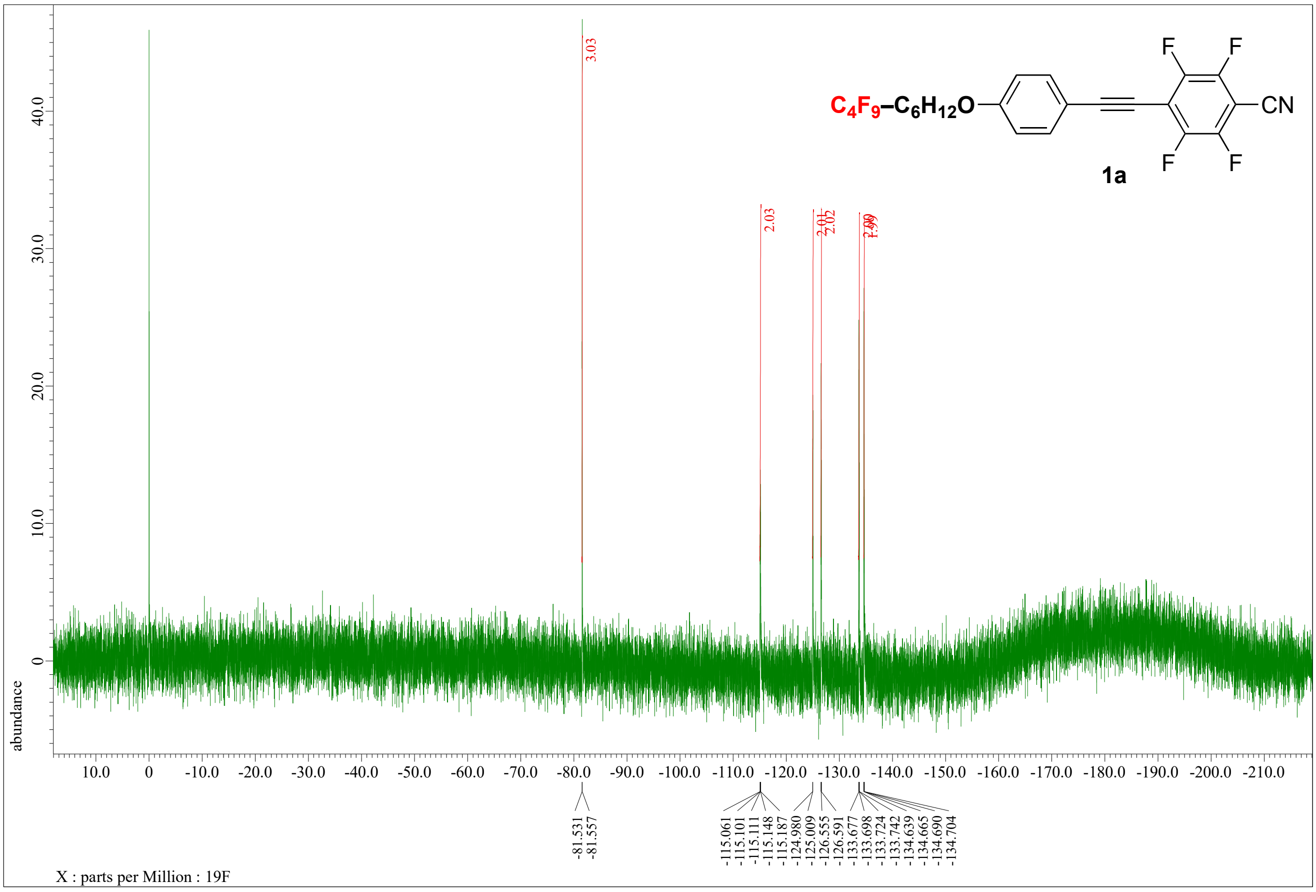


**Figure S3.** $^{19}$F NMR spectrum of **1a** ($CDCl_3$, $CFCl_3$, 376 MHz).

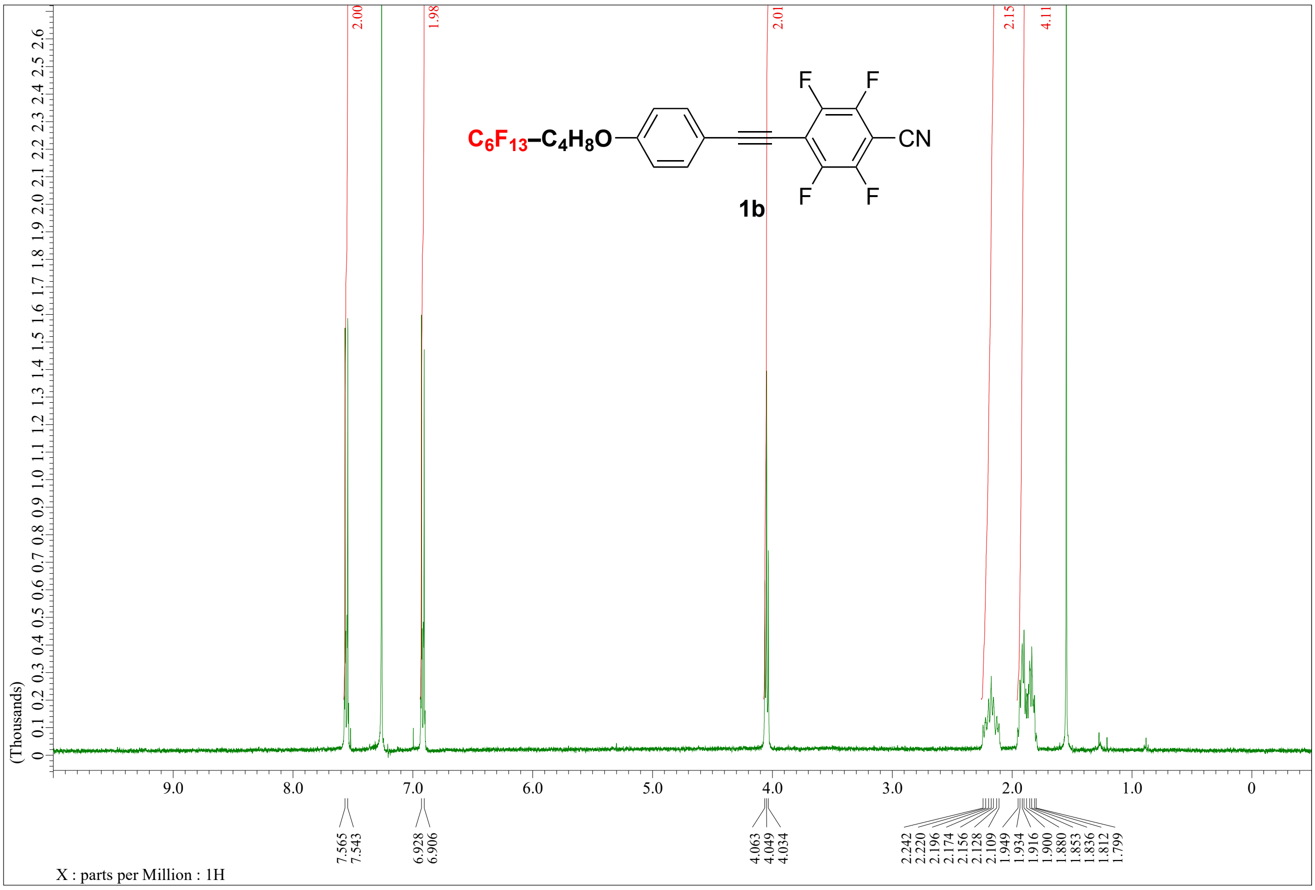


**Figure S4.** $^{1}$H NMR spectrum of **1b** ($CDCl_3$, 400 MHz).

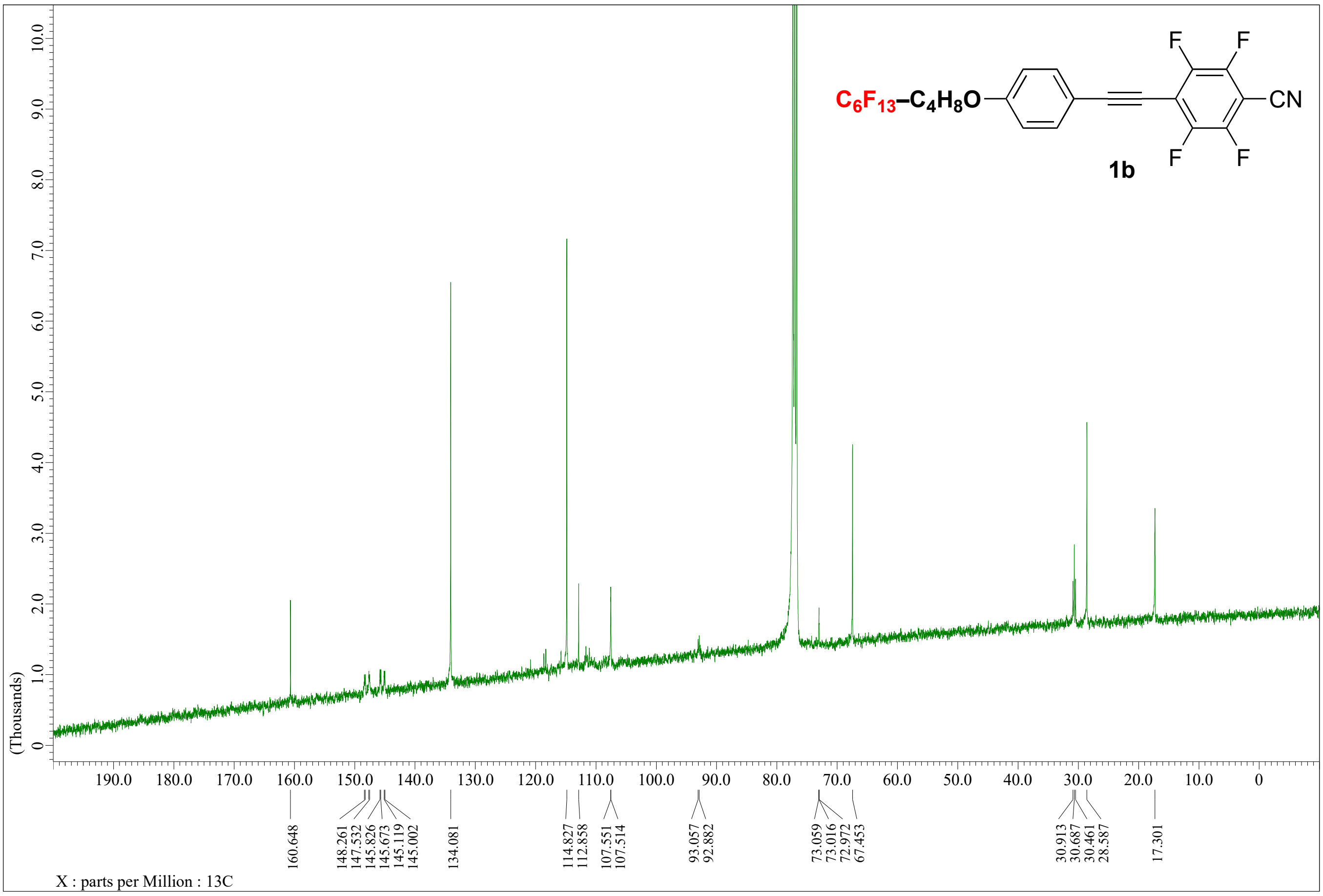


**Figure S5.** $^{13}$C NMR spectrum of **1b** ($CDCl_3$, 100 MHz).

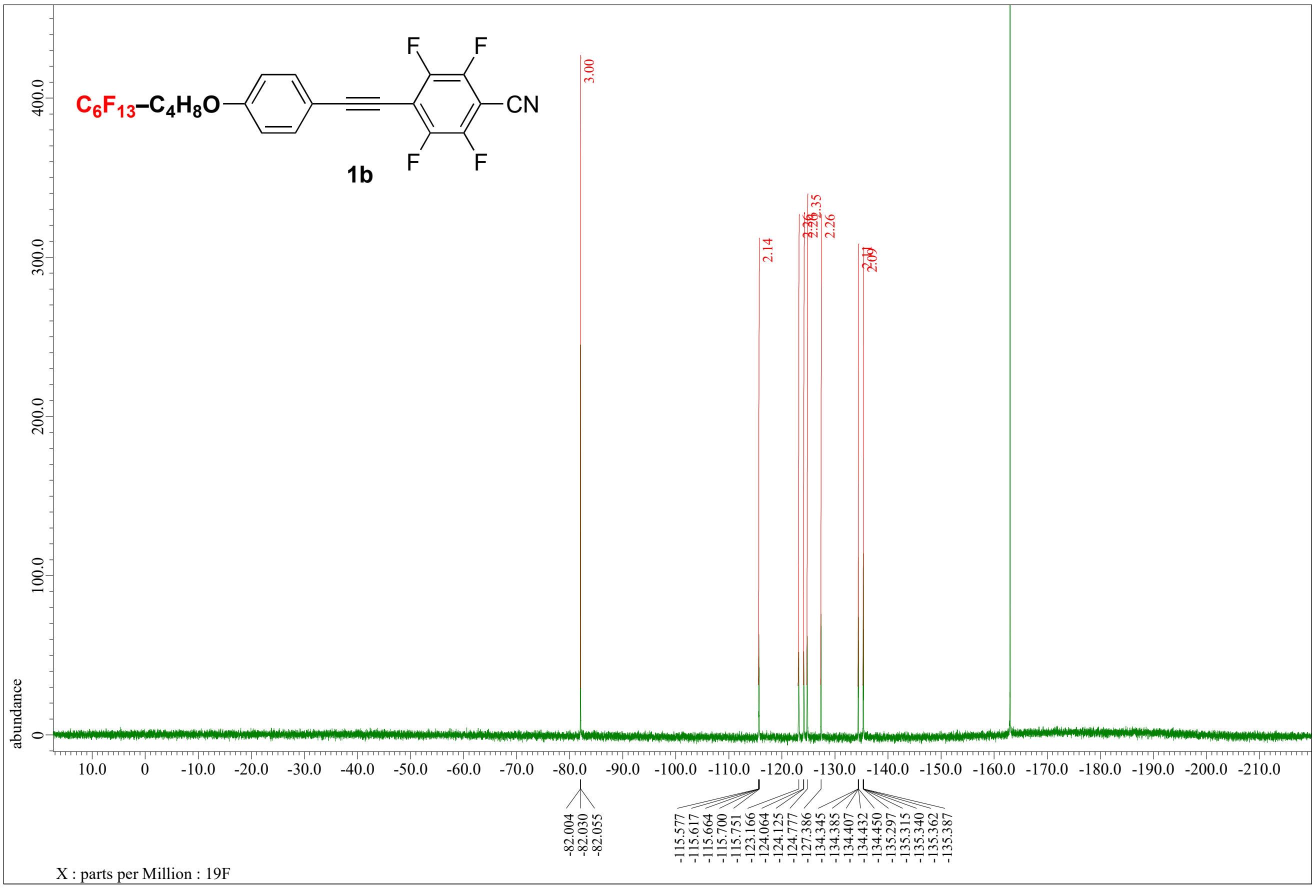


**Figure S6.** $^{19}F$ NMR spectrum of **1b** ($CDCl_3$, $C_6F_6$, 376 MHz).

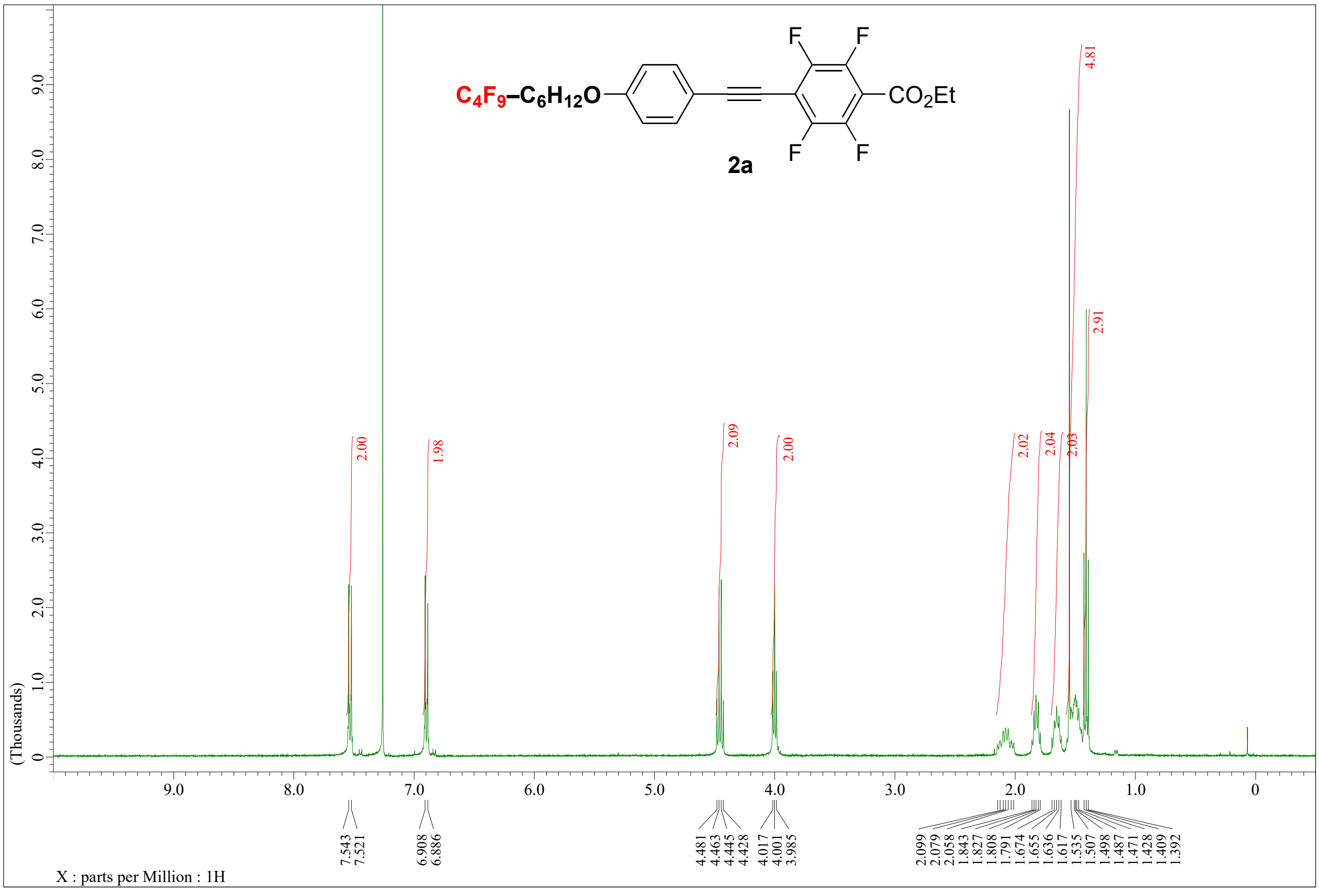


**Figure S7.** $^{1}$H NMR spectrum of **2a** ($CDCl_3$, 400 MHz).

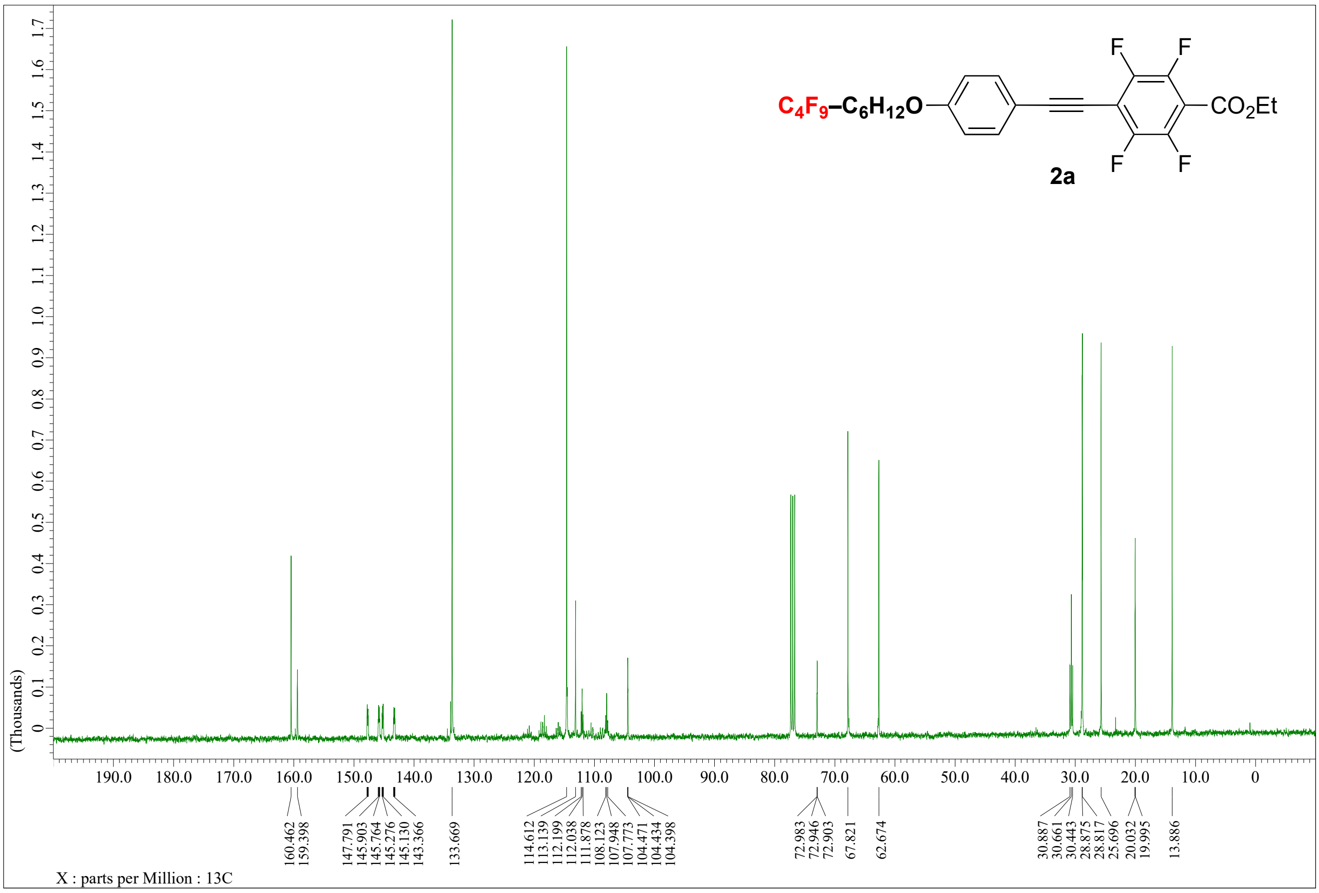


**Figure S8.** $^{13}$C NMR spectrum of **2a** ($CDCl_3$, 100 MHz).

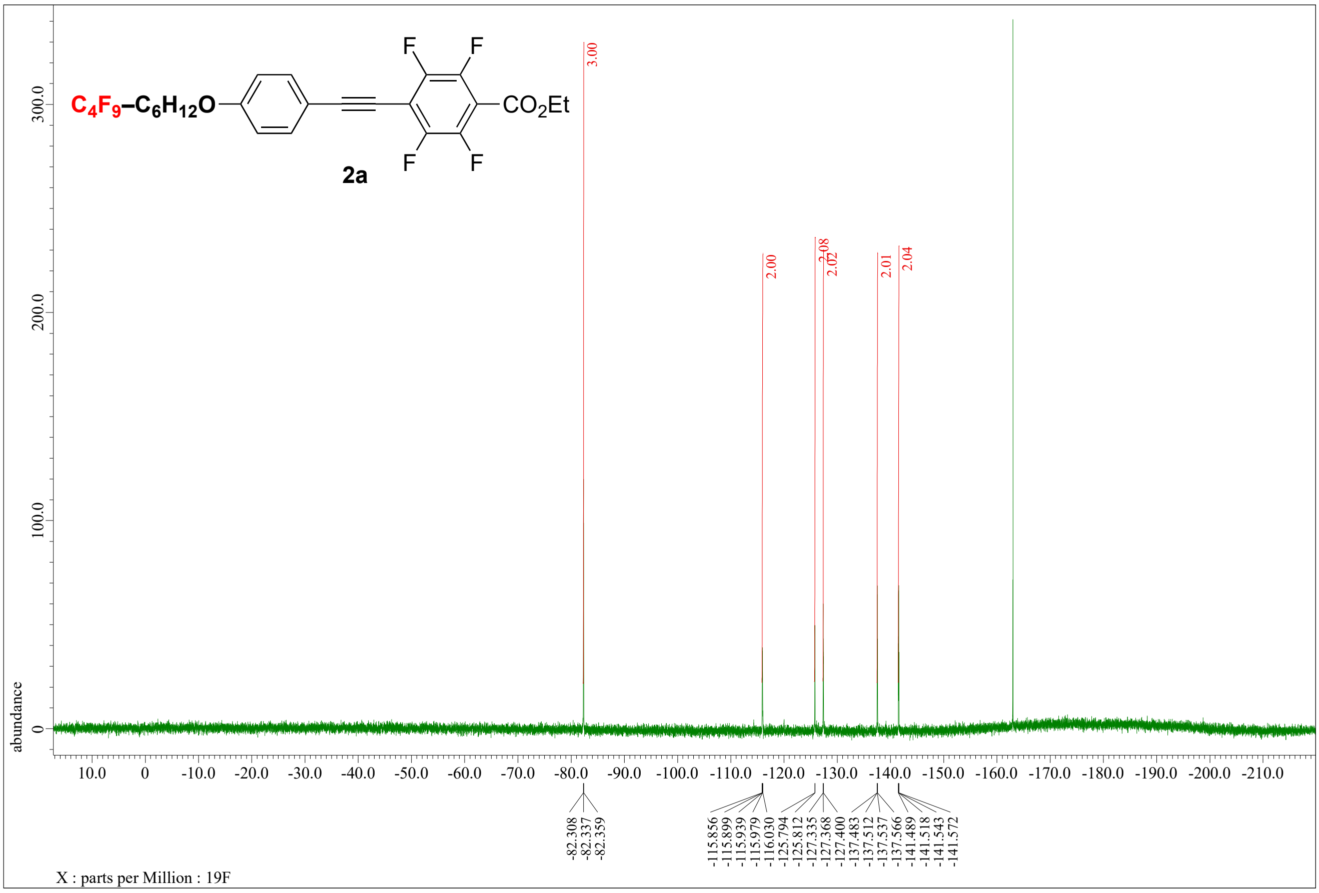


**Figure S9.** $^{19}$F NMR spectrum of **2a** ($CDCl_3$, $C_6F_6$, 376 MHz).

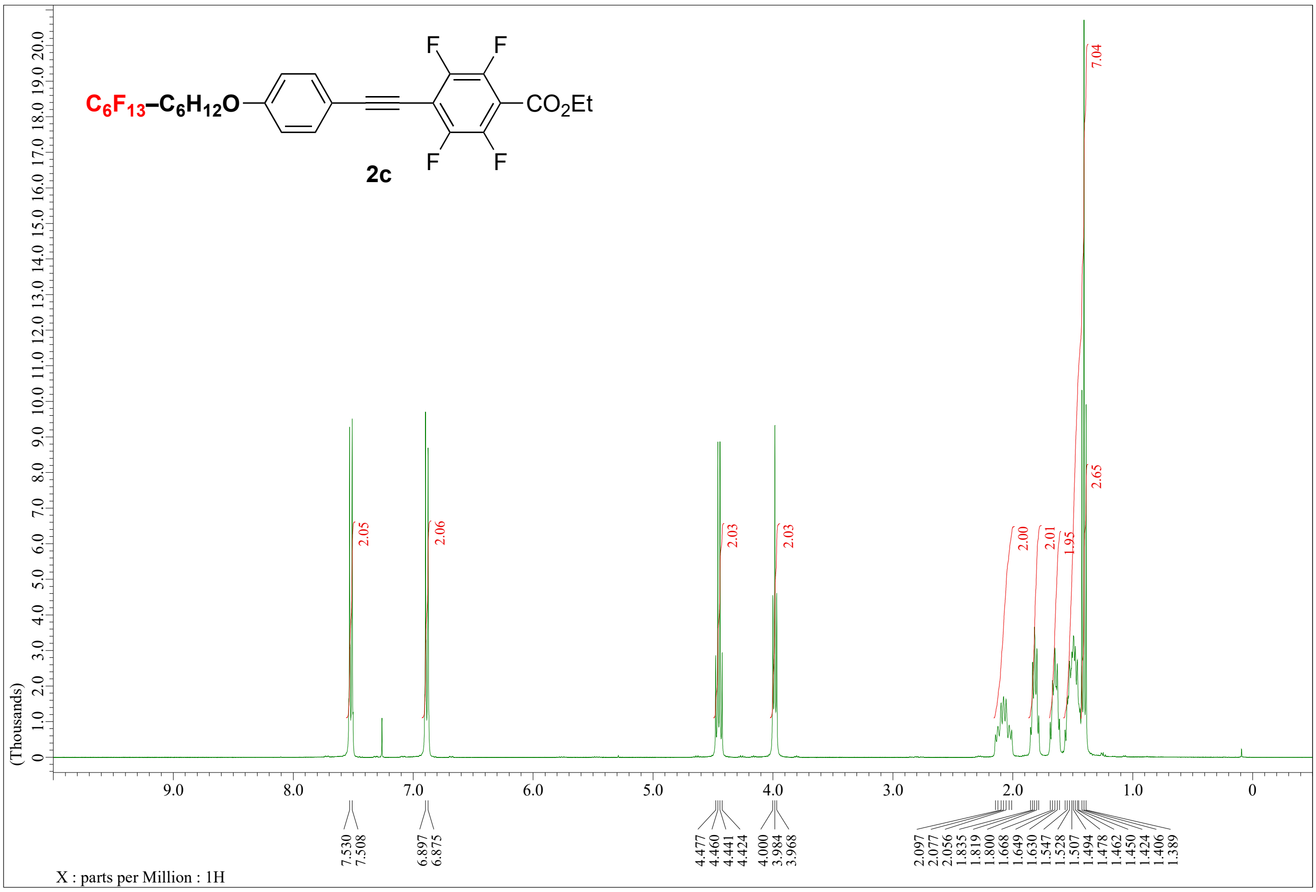

**Figure S10.** $^{1}$H NMR spectrum of **2c** ($CDCl_3$, 400 MHz).

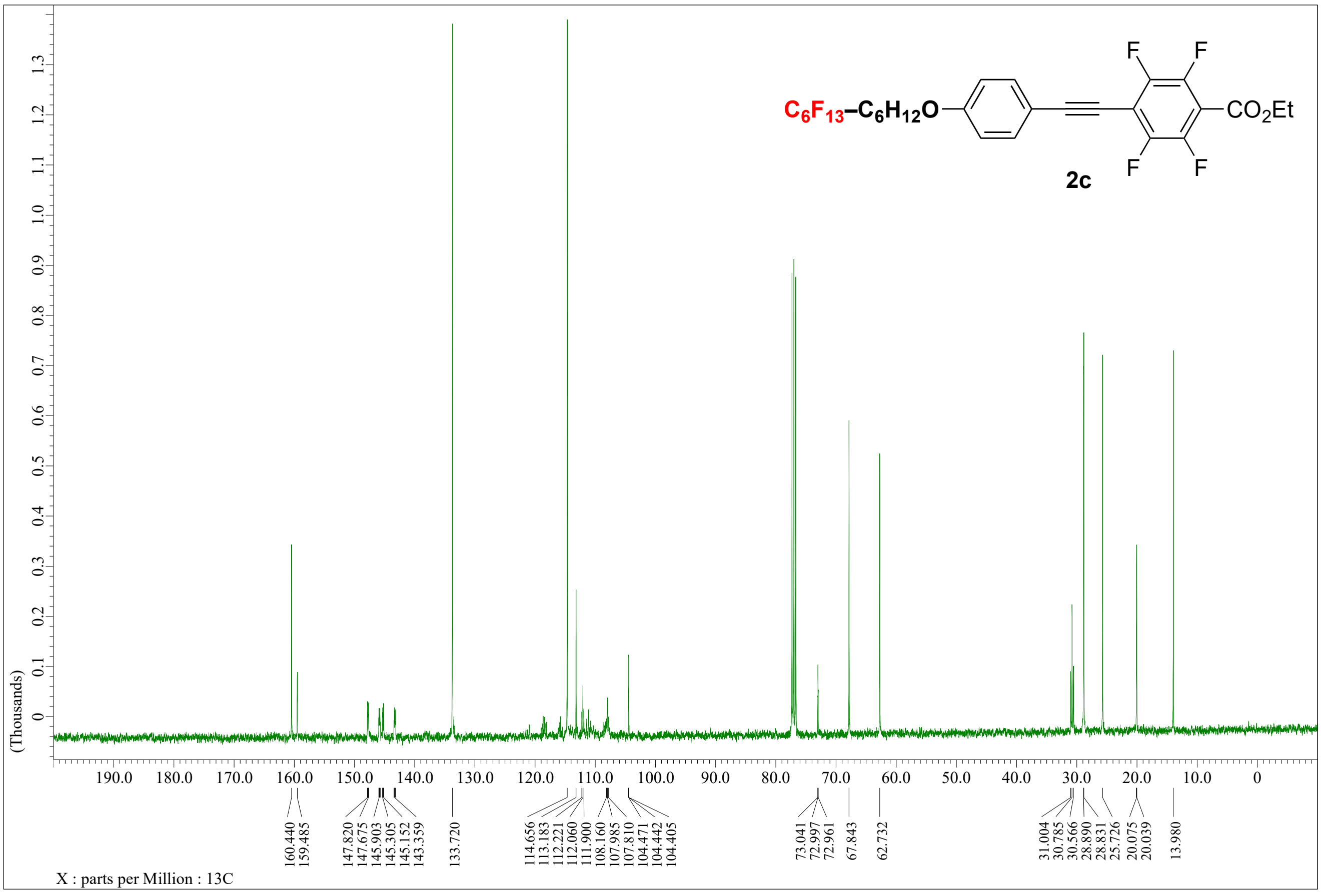

**Figure S11.** $^{13}$C NMR spectrum of **2c** ($CDCl_3$, 100 MHz).

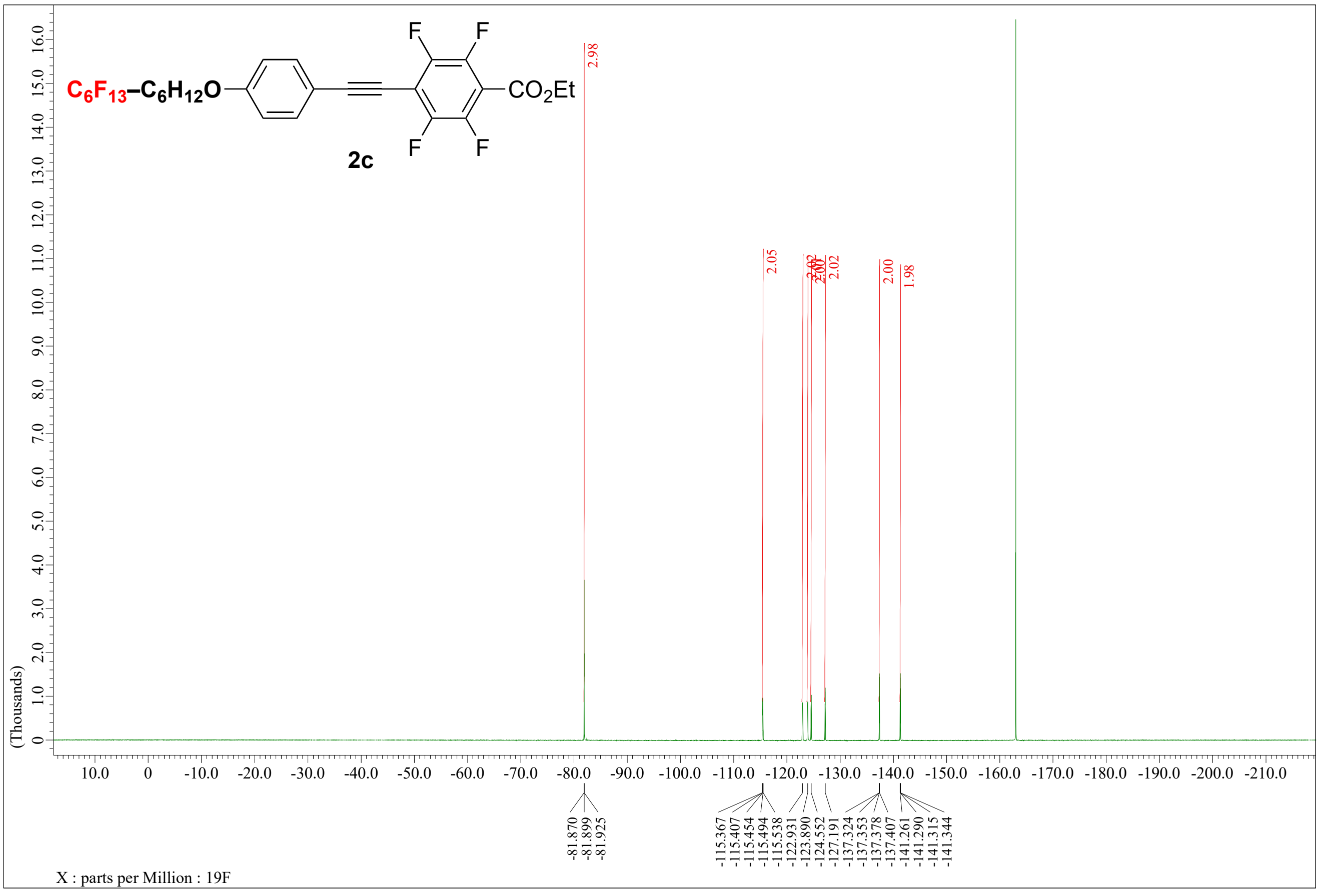


**Figure S12.** $^{19}$F NMR spectrum of **2c** ($CDCl_3$, $C_6F_6$, 376 MHz).

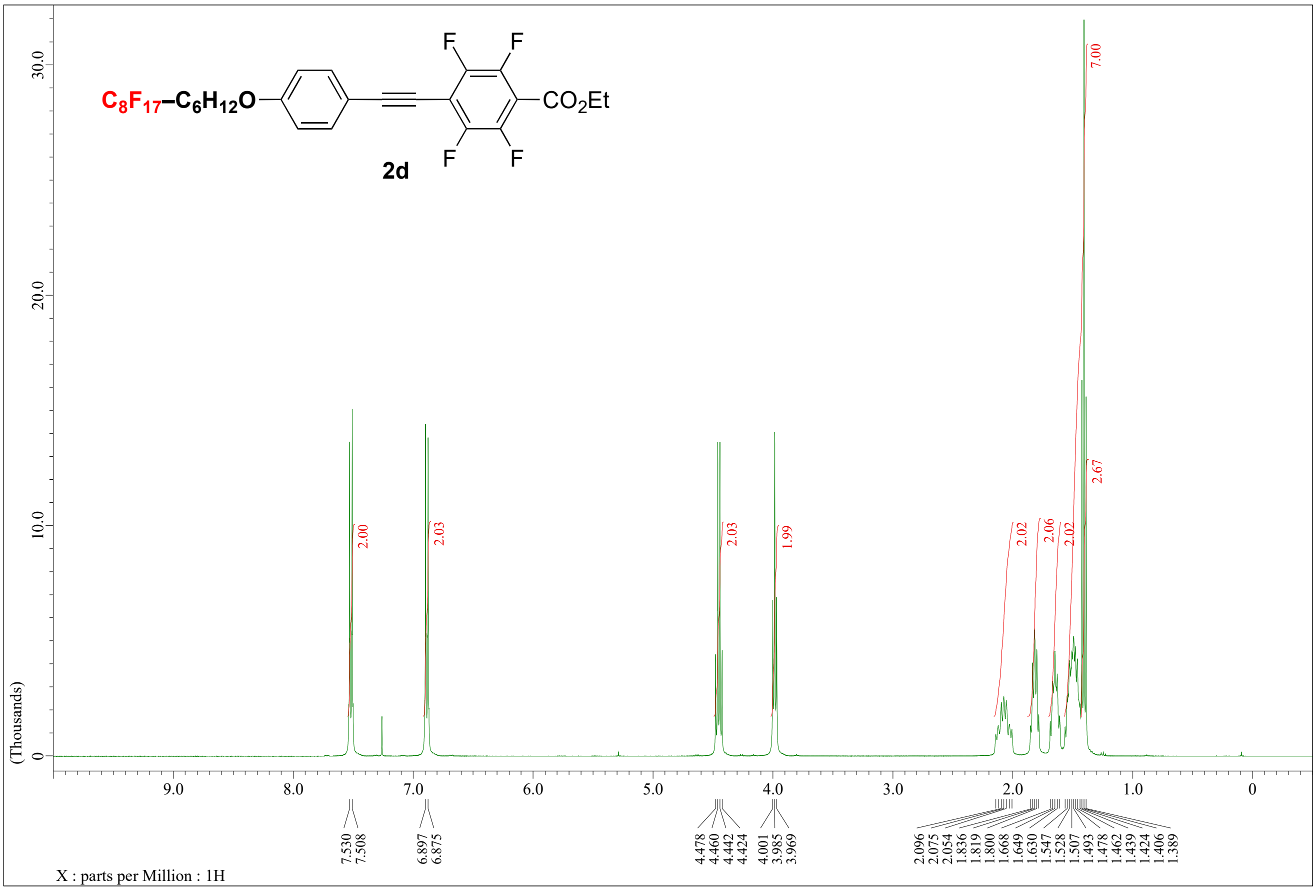


**Figure S13.** $^1$H NMR spectrum of **2d** ($CDCl_3$, 400 MHz).

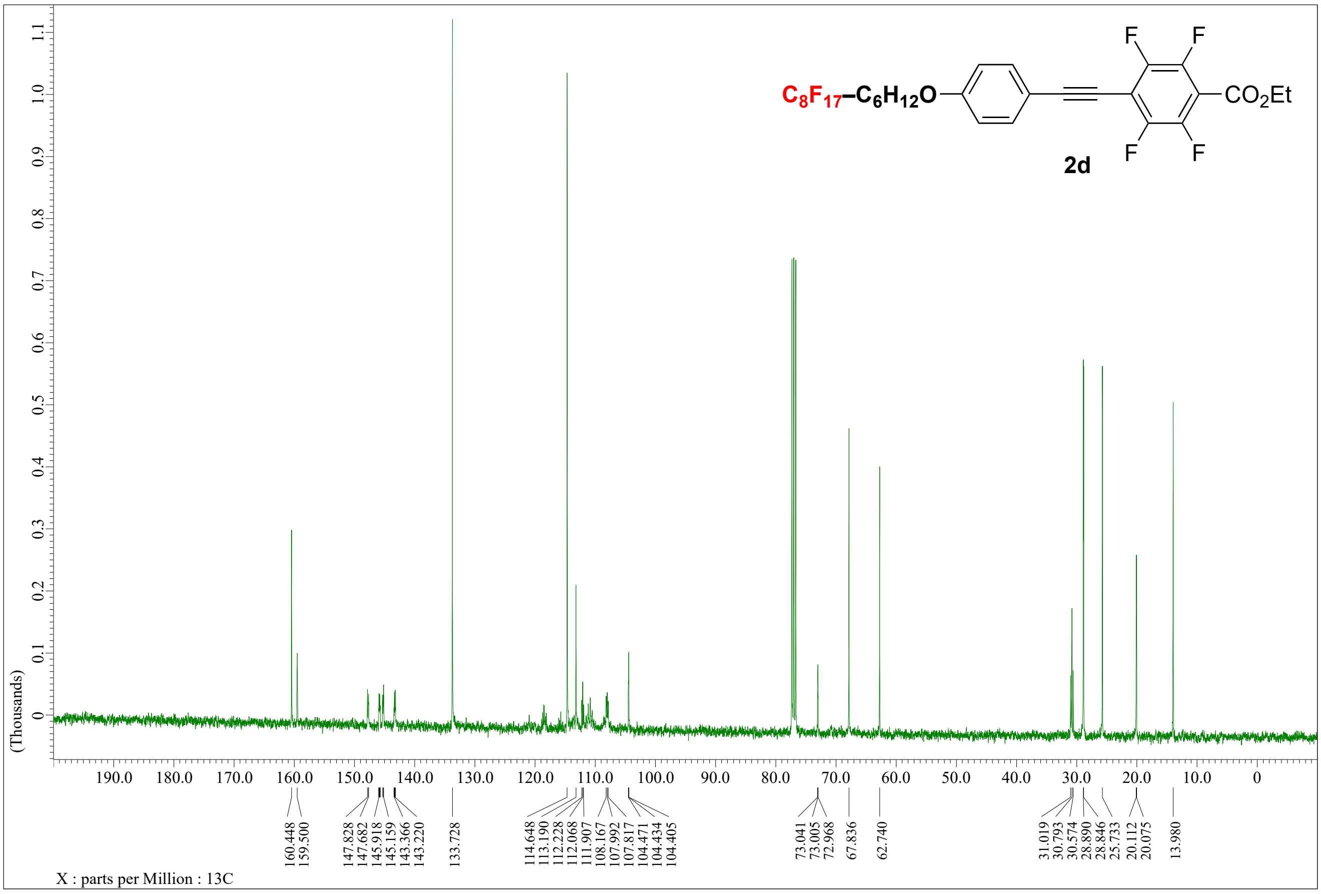


**Figure S14.** $^{13}$C NMR spectrum of **2d** ($CDCl_3$, 100 MHz).

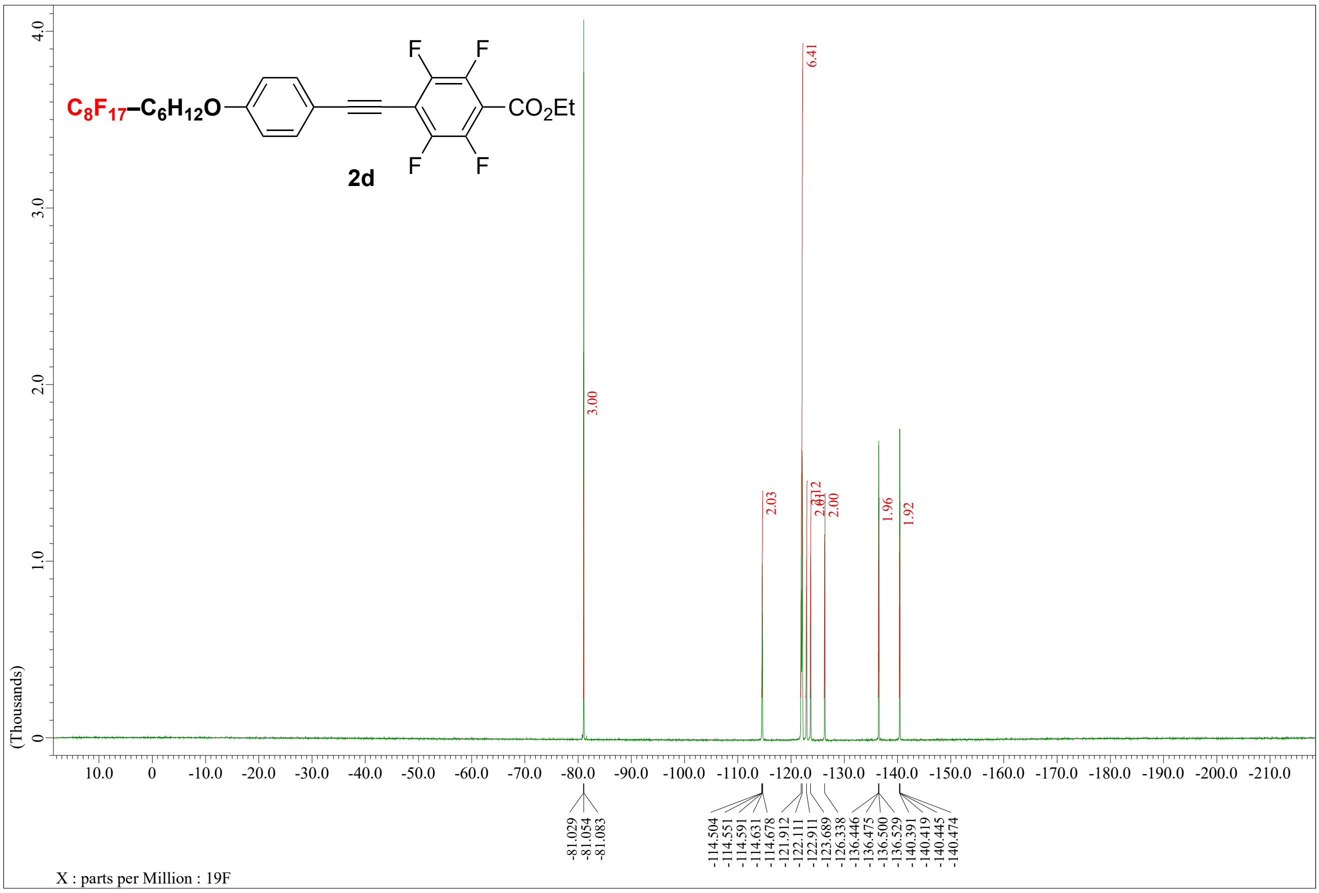


**Figure S15.** $^{19}$F NMR spectrum of **2d** ($CDCl_3$, $CFCl_3$, 376 MHz).

**Phase transition behavior**

(a) **1a**

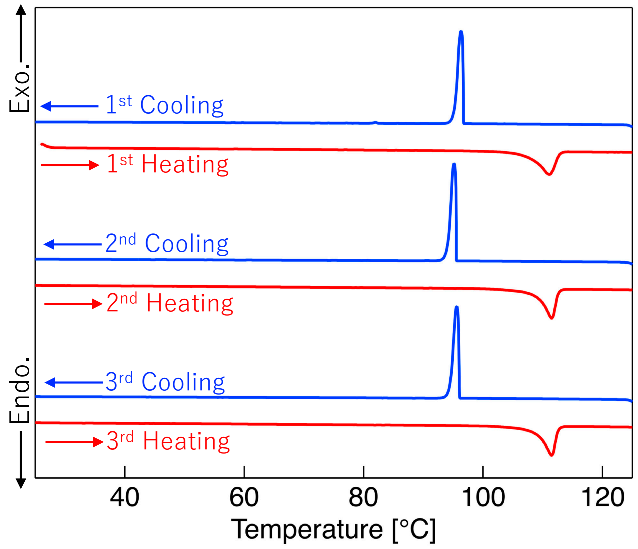


(b) **1b**

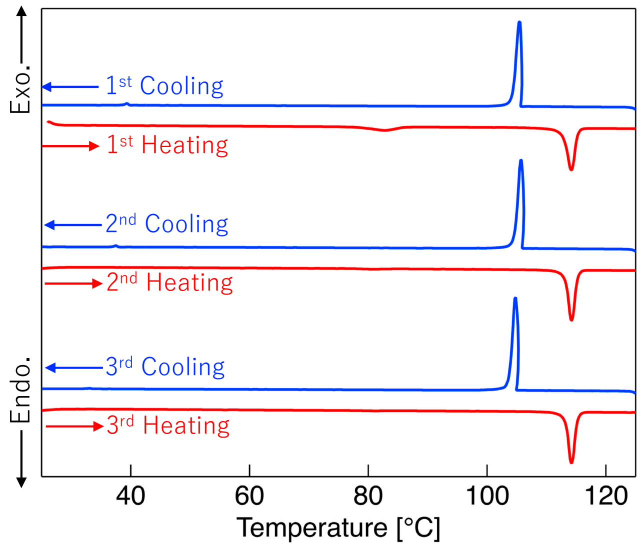


(c) **2a**

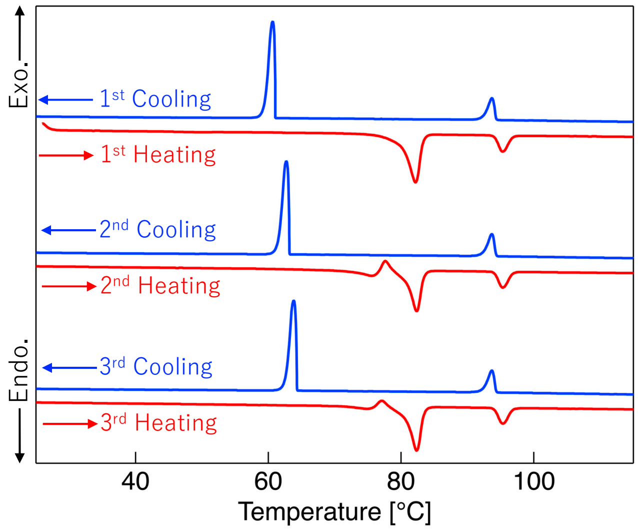


(d) **2c**

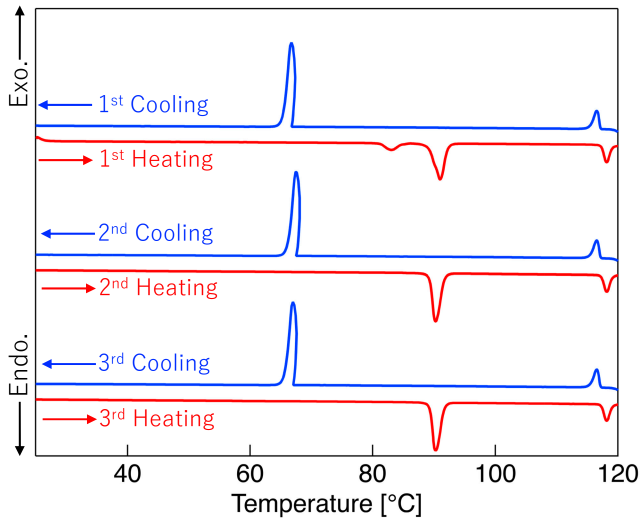


(e) **2d**

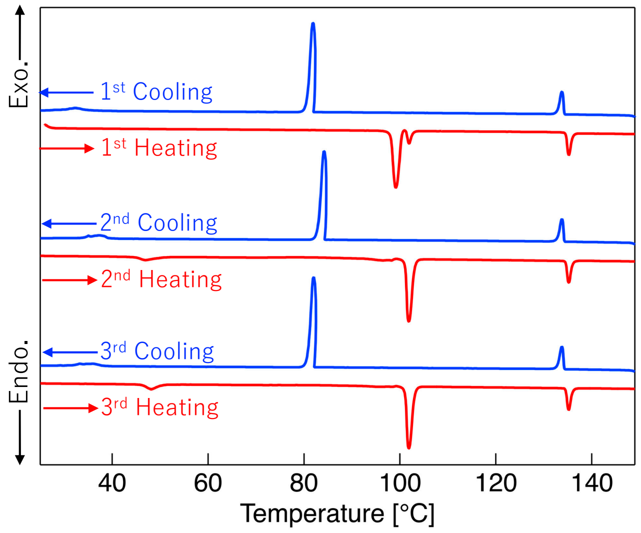


**Figure S16.** DSC Thermograms of **1a, 1b, 2a, 2c, and 2d** under $N_2$ atmosphere. Scan rate: 5.0 °C min$^{-1}$.

(a) **2a** at 84 °C (b) **2c** at 110 °C (c) **2d** at 130 °C

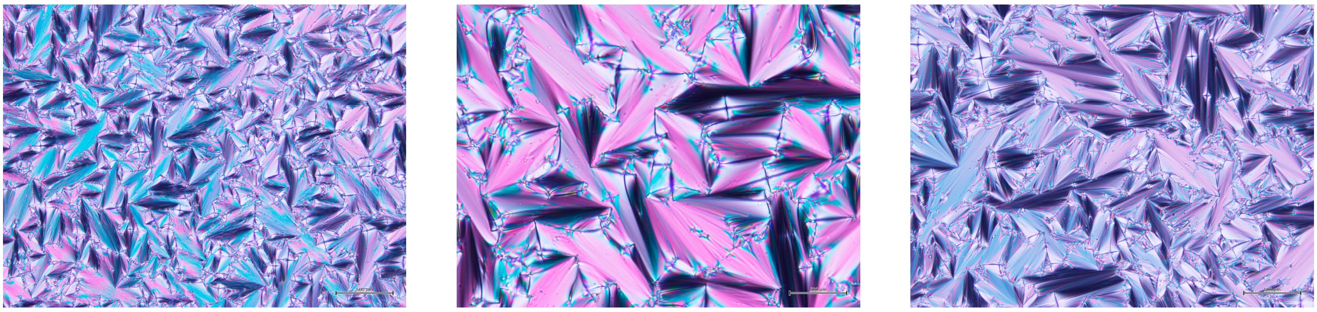

**Figure S17.** Optical texture of (a) **2a**, (b) **2c**, and **2d** observed by POM.

**Table S1.** Phase transition behavior of **1a, 1b, 2a, 2c, and 2d** observed by DSC measurement.

| Molecule | Phase sequence | | Temperature [°C] | Enthalpy ($\Delta H$) [kJ mol$^{-1}$] | Entropy ($\Delta S$) [J mol$^{-1}$ K$^{-1}$] |
|---|---|---|---|---|---|
| **1a** | 1$^{st}$ Heating | Cry-Iso | 108 | 29.6 | 77.6 |
| | 1$^{st}$ Cooling | Iso-Cry | 97 | -27.7 | -75.1 |
| | 2$^{nd}$ Heating | Cry-Iso | 109 | 28.6 | 75.0 |
| | 2$^{nd}$ Cooling | Iso-Cry | 96 | -28.1 | -76.2 |
| | 3$^{rd}$ Heating | Cry-Iso | 109 | 28.8 | 75.5 |
| | 3$^{rd}$ Cooling | Iso-Cry | 96 | -28.2 | -76.4 |
| **1b** | 1$^{st}$ Heating | Cry$^1$-Cry$^2$ | 79 | 5.02 | 14.3 |
| | | Cry$^2$-Iso | 113 | 22.1 | 57.2 |
| | 1$^{st}$ Cooling | Iso-Cry | 106 | -20.7 | -54.7 |
| | 2$^{nd}$ Heating | Cry-Iso | 113 | 21.6 | 55.9 |
| | 2$^{nd}$ Cooling | Iso-Cry | 106 | -20.7 | -54.6 |
| | 3$^{rd}$ Heating | Cry-Iso | 113 | 23.7 | 61.3 |
| | 3$^{rd}$ Cooling | Iso-Cry | 105 | -20.8 | -55.1 |
| **2a** | 1$^{st}$ Heating | Cry-SmA | 80 | 29.7 | 84.1 |
| | | SmA-Iso | 94 | 6.74 | 18.4 |
| | 1$^{st}$ Cooling | Iso-SmA | 95 | -6.69 | -18.2 |
| | | SmA-Cry | 61 | -21.9 | -65.5 |
| | 2$^{nd}$ Heating | Cry$^1$-Cry$^2$ | 77 | 19.3 | 55.2 |
| | | Cry$^2$-SmA | 80 | 22.4 | 63.3 |
| | | SmA-Iso | 94 | 6.72 | 18.3 |
| | 2$^{nd}$ Cooling | Iso-SmA | 94 | -6.71 | -18.3 |
| | | SmA-Cry$^1$ | 63 | -21.4 | -63.7 |
| | 3$^{rd}$ Heating | Cry$^1$-Cry$^2$ | 76 | 6.61 | 19.0 |
| | | Cry$^2$-SmA | 80 | 22.5 | 63.6 |
| | | SmA-Iso | 94 | 6.76 | 18.4 |
| | 3$^{rd}$ Cooling | Iso-SmA | 94 | -6.64 | -18.1 |
| | | SmA-Cry$^1$ | 64 | -21.8 | -64.6 |

**Table S1 (continue).** Phase transition behavior of **1a, 1b, 2a, 2c, and 2d** observed by DSC measurement.

| Molecule | Phase sequence | | Temperature [°C] | Enthalpy (*ΔH*) [kJ mol$^{-1}$] | Entropy (*ΔS*) [J mol$^{-1}$ K$^{-1}$] |
|---|---|---|---|---|---|
| **2c** | 1$^{st}$ Heating | Cry$^1$-Cry$^2$ | 81 | 6.43 | 18.2 |
| | | Cry$^2$-SmA | 89 | 29.3 | 80.9 |
| | | SmA-Iso | 117 | 8.31 | 21.3 |
| | 1$^{st}$ Cooling | Iso-SmA | 117 | -8.29 | -21.2 |
| | | SmA-Cry$^1$ | 67 | -27.6 | -81.1 |
| | 2$^{nd}$ Heating | Cry-SmA | 89 | 29.8 | 82.3 |
| | | SmA-Iso | 117 | 8.22 | 21.1 |
| | 2$^{nd}$ Cooling | Iso-SmA | 117 | -8.3 | -21.3 |
| | | SmA-Cry | 67 | -27.7 | -81.3 |
| | 3$^{rd}$ Heating | Cry-SmA | 89 | 29.7 | 81.8 |
| | | SmA-Iso | 117 | 8.57 | 21.9 |
| | 3$^{rd}$ Cooling | Iso-SmA | 117 | -8.40 | -21.5 |
| | | SmA-Cry | 67 | -27.5 | -81.0 |
| **2d** | 1$^{st}$ Heating | Cry-SmA | 98 | 39.2 | 105.6 |
| | | SmA-Iso | 135 | 10.6 | 26.0 |
| | 1$^{st}$ Cooling | Iso-SmA | 134 | -10.2 | -25.0 |
| | | SmA-Cry$^2$ | 82 | -34.6 | -97.7 |
| | | Cry$^2$-Cry$^1$ | 35 | -5.64 | -18.3 |
| | 2$^{nd}$ Heating | Cry$^1$-Cry$^2$ | 45 | 5.29 | 16.6 |
| | | Cry$^2$-SmA | 101 | 38.0 | 101.6 |
| | | SmA-Iso | 135 | 10.1 | 24.8 |
| | 2$^{nd}$ Cooling | Iso-SmA | 134 | -10.1 | -24.9 |
| | | SmA-Cry$^2$ | 84 | -35.1 | -98.1 |
| | | Cry$^2$-Cry$^1$ | 39 | -5.39 | -17.3 |
| | 3$^{rd}$ Heating | Cry$^1$-Cry$^2$ | 46 | 6.05 | 18.9 |
| | | Cry$^2$-SmA | 101 | 38.1 | 101.7 |
| | | SmA-Iso | 135 | 9.82 | 24.1 |
| | 3$^{rd}$ Cooling | Iso-SmA | 134 | -10.2 | -25.1 |
| | | SmA-Cry$^2$ | 82 | -34.7 | -97.6 |
| | | Cry$^2$-Cry$^1$ | 38 | -5.13 | -16.5 |

## Varied Temperature Powder X-ray Diffraction (VT-PXRD)

(a) **2a** in SmA phase at 80 °C

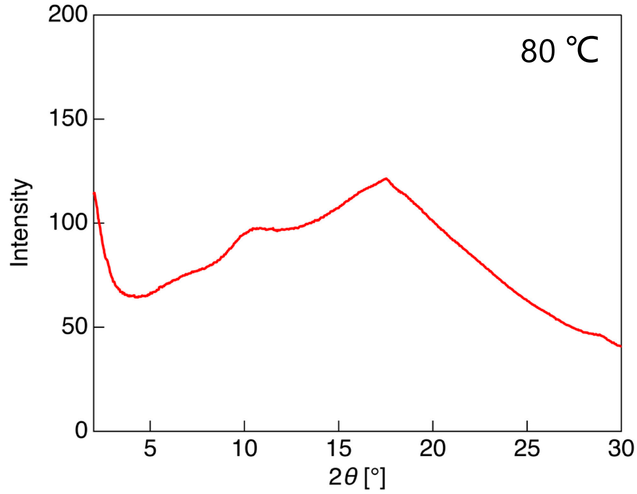


(b) **2a** in Cry phase at 40 °C

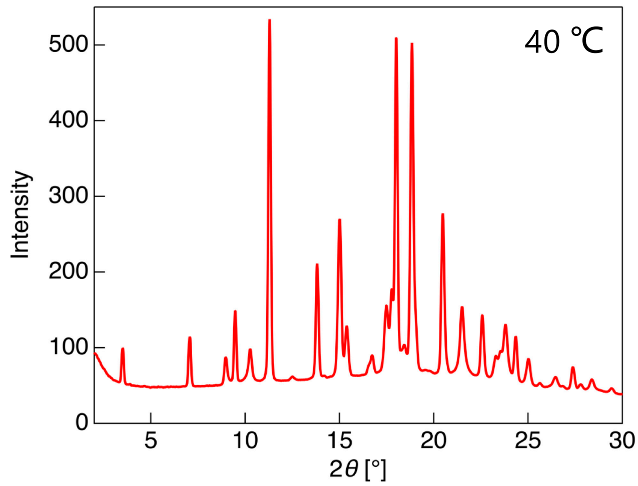


(c) **2c** in SmA phase at 90 °C

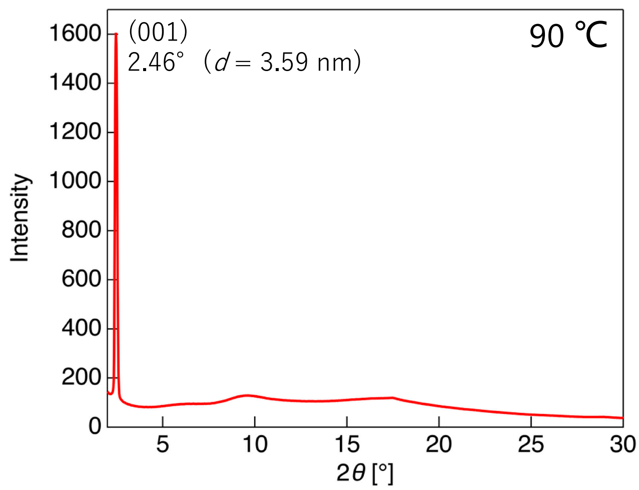


(d) **2c** in Cry phase at 40 °C

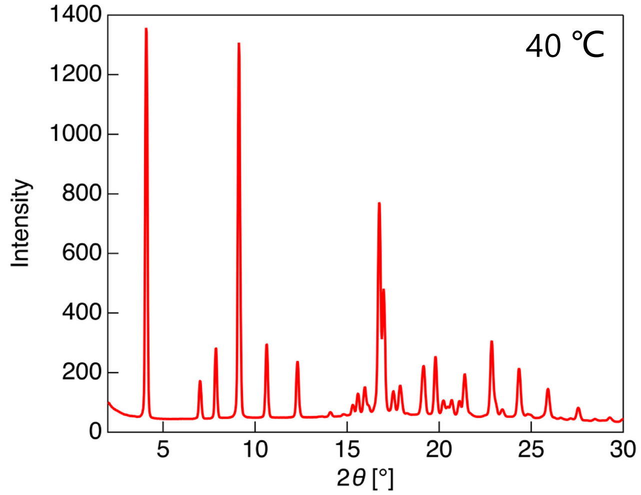


(e) **2d** in SmA phase at 110 °C

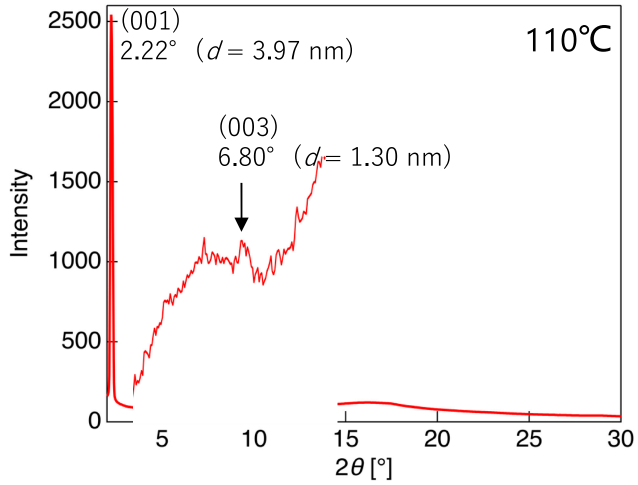


(f) **2d** in Cry phase at 40 °C

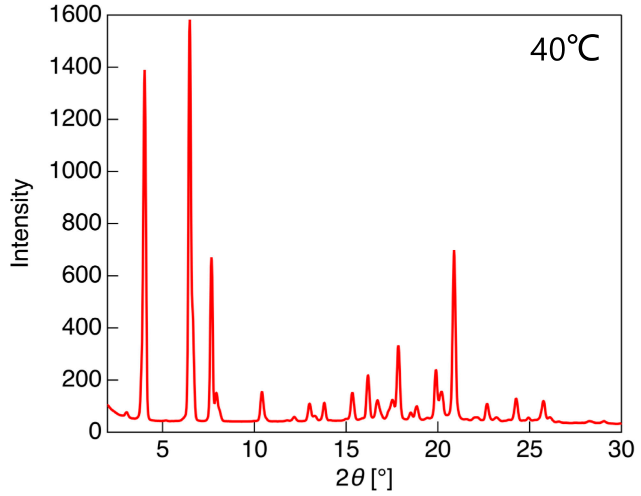


**Figure S18.** VT-PXRD patterns of (a) **2a** in SmA phase at 80 °C, (b) **2a** in Cry phase at 40 °C, (c) **2c** in SmA phase at 90 °C, (d) **2c** in Cry phase at 40 °C, (e) 2d in SmA phase at 110 °C, and (f) **2d** in Cry phase at 40 °C.

## Photophysical behavior

## $CH_2Cl_2$ solution phase

(a) **1a**

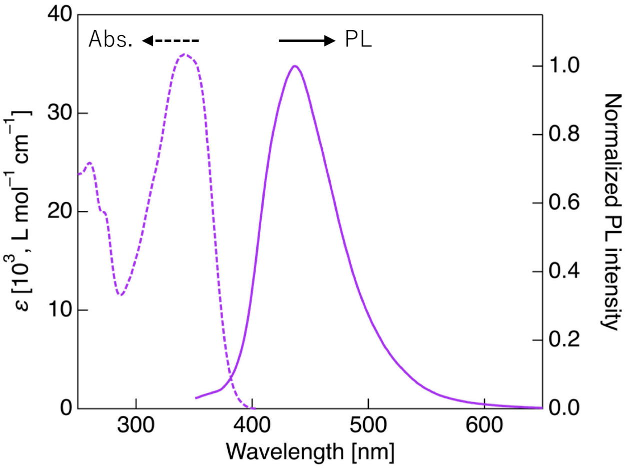


(b) **1b**

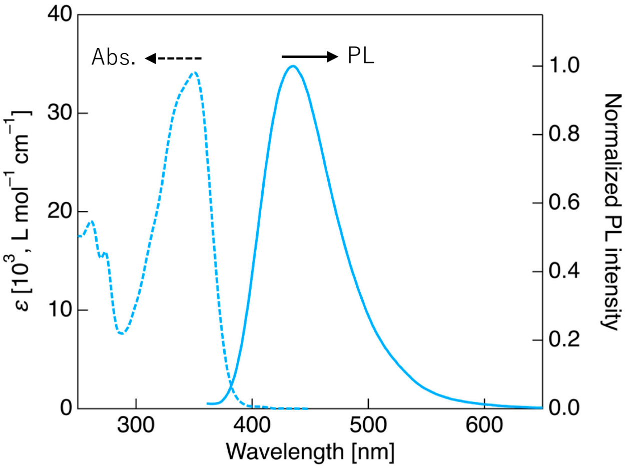


(c) **2a**

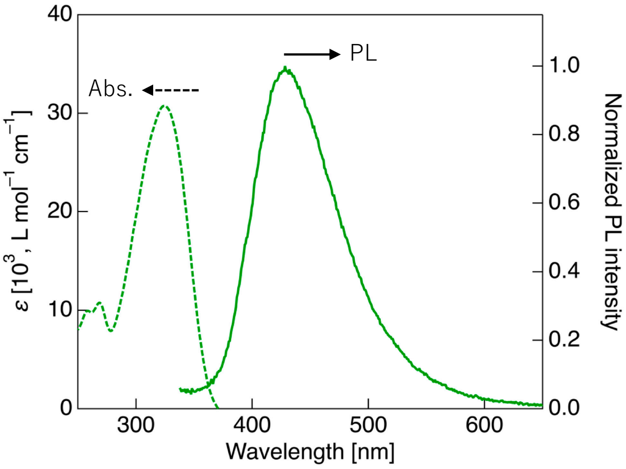


(d) **2c**

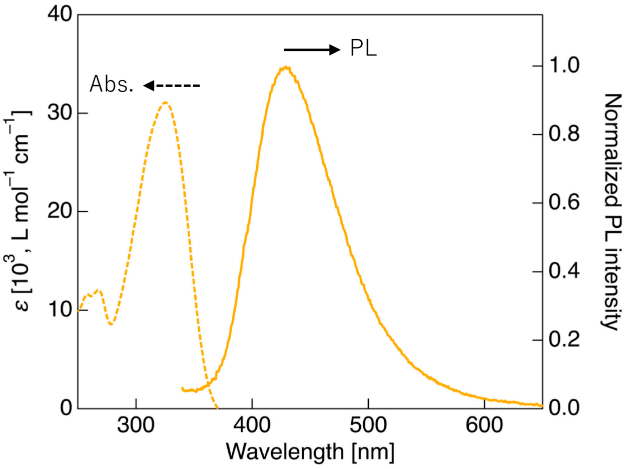


(e) **2d**

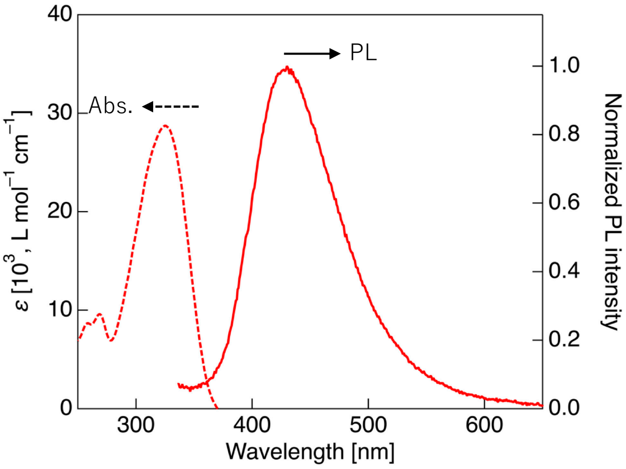


**Figure S19.** UV-vis absorption (1.0 x $10^{-5}$ mol $L^{-1}$) and PL spectrum (1.0 x $10^{-6}$ mol $L^{-1}$) of (a) **1a**, (b) **1b**, (c) **2a**, (d) **2c**, and (e) **2d** in $CH_2Cl_2$ solution. Excitation wavelength: $\lambda_{abs}$ at the long-wavelength side.

## Cry phase

(a) **1a**

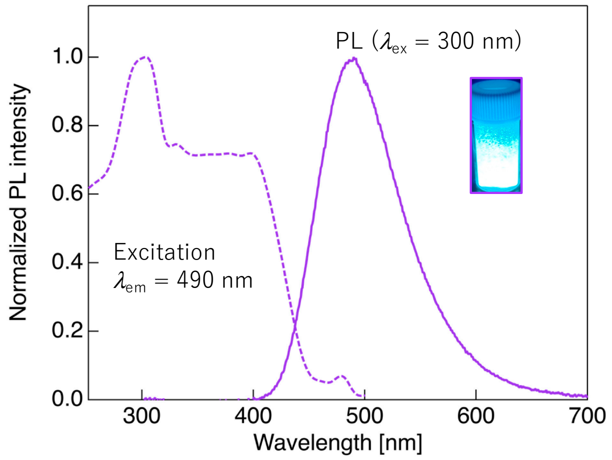


(b) **1b**

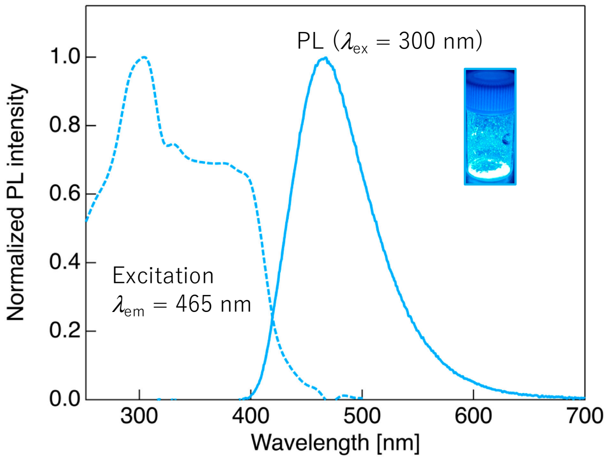


(c) **1c**

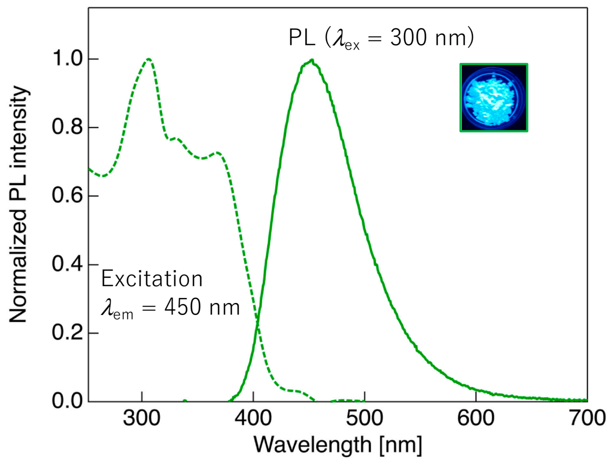


(d) **1d**

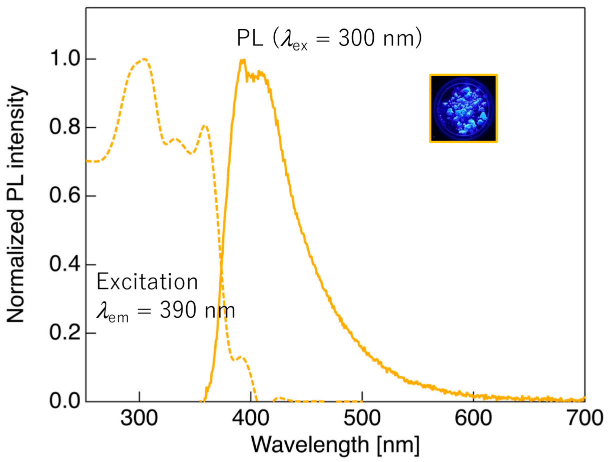


(e) **1e**

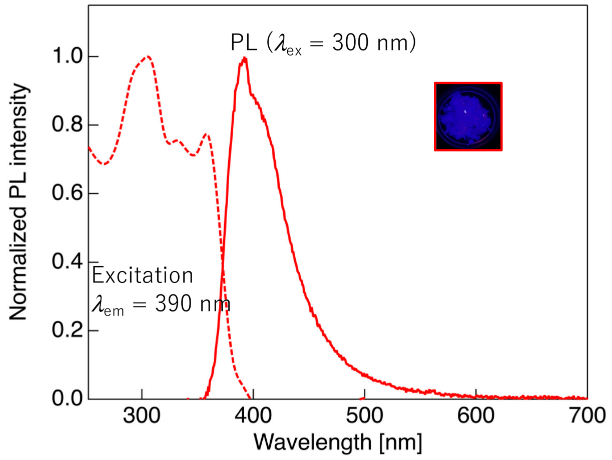


**Figure S20.** Excitation and PL spectra of (a) **1a**, (b) **1b**, (c) **2a**, (d) **2c**, and (e) **2d** in Cry phase. Measured using a PL quantum yield measurement system.

**PXRD Measurement at 25 °C**

(a) **1a**

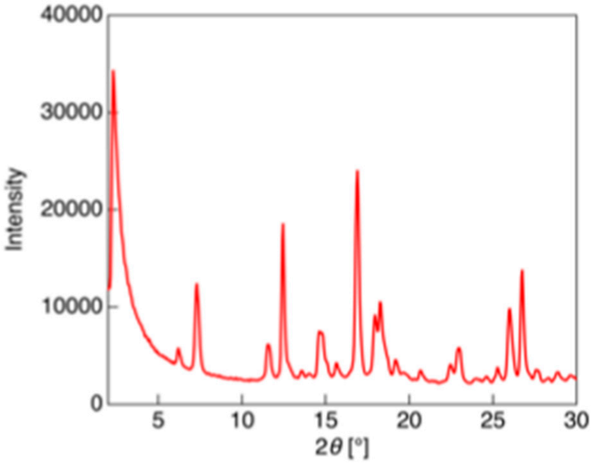


(b) **1b**

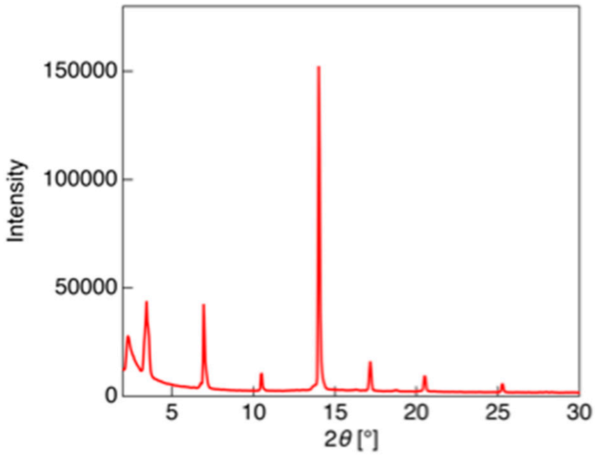


(c) **2a**

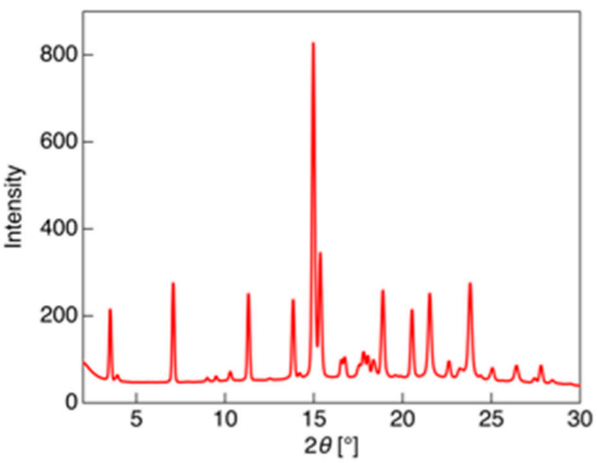


(d) **2c**

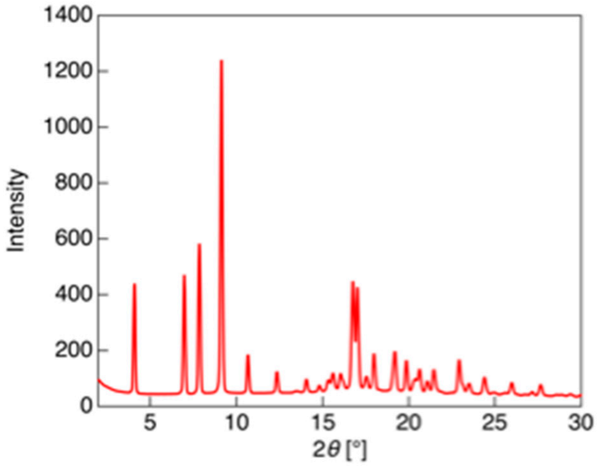


(e) **2d**

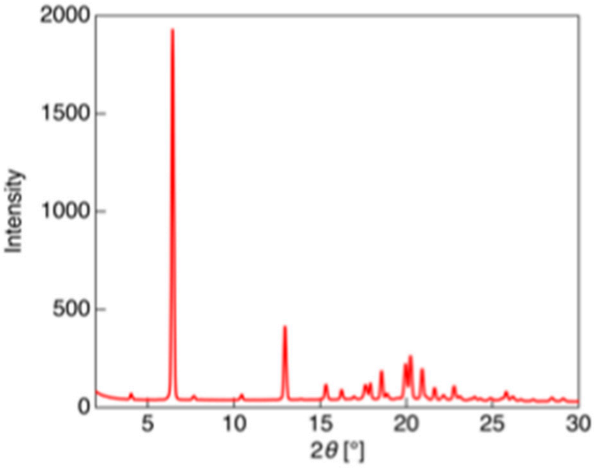


**Figure S21.** PXRD patterns of (a) **1a**, (b) **1b**, (c) **2a**, (d) **2c**, and (e) **2d** measured at 25 °C.

## Varied-temperature PL behavior

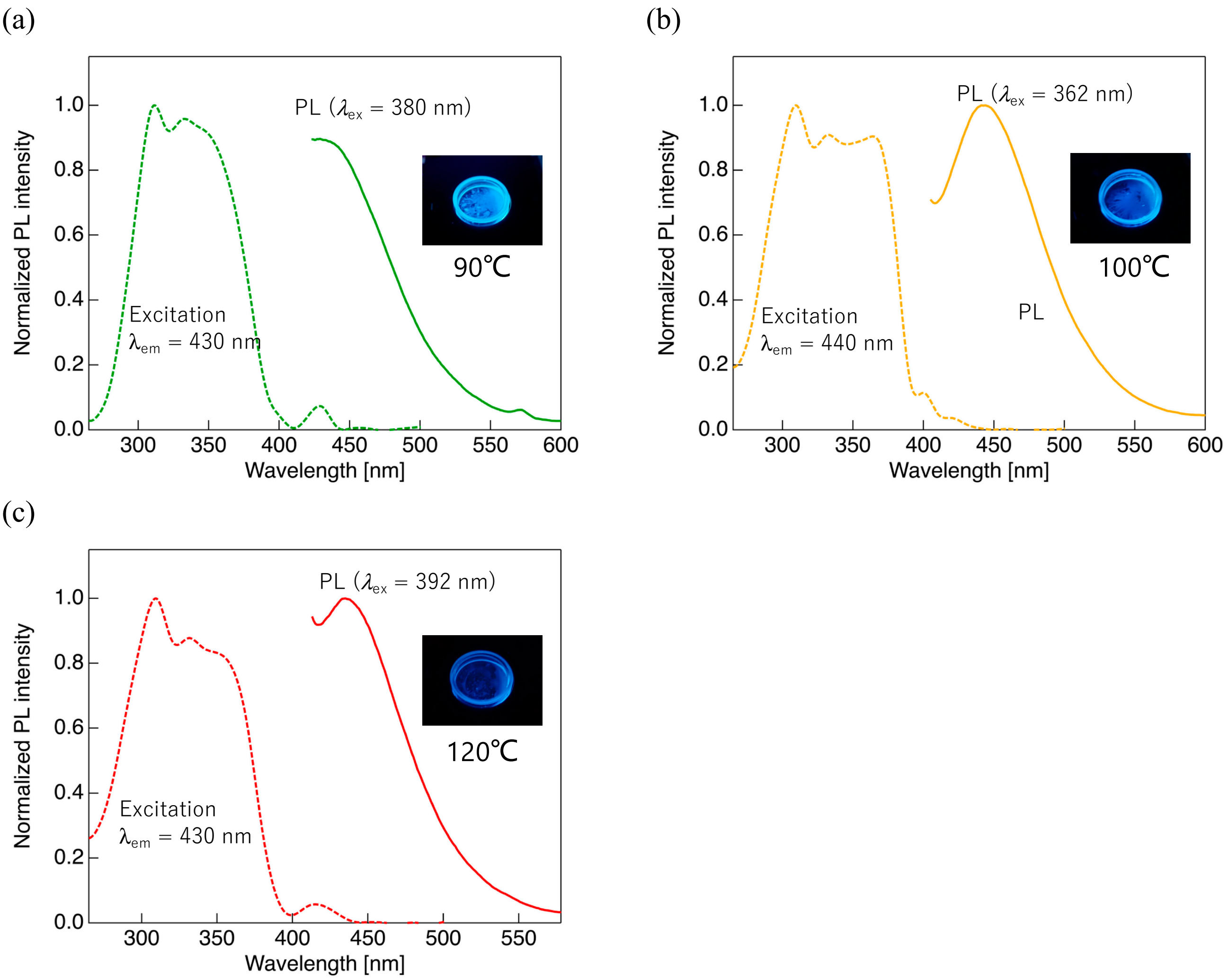


**Figure S22.** (a) Excitation and PL spectrum of (a) **2a** (90 °C), (b) **2c** (100 °C), and (c) **2d** (120 °C).

## Quantum chemical calculation

Quantum chemical calculation was performed using the Gaussian 16 program set. The initial structure for quantum chemical calculations was optimized by the structural optimization calculation with the M06-2X functional and the 6-31+G(d,p) basis set. Theoretical vertical electronic transitions were determined by time-dependent self-consistent field (TD-SCF) approximation.

(a) Optimized structure

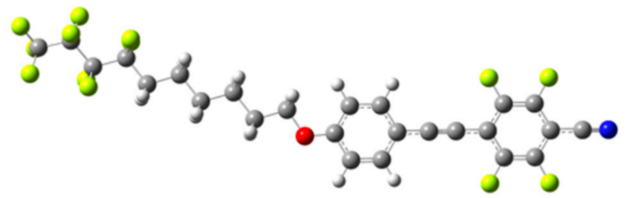

(b) HOMO–2 (–9.07 eV)

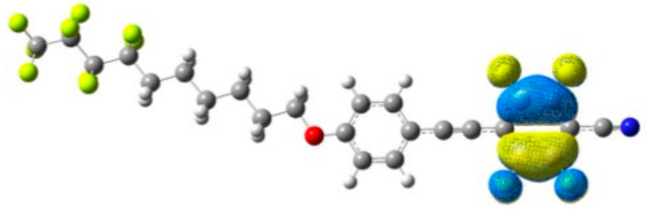

(c) HOMO (–7.50 eV)

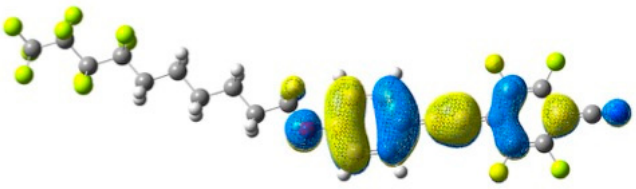

(d) LUMO (–1.96 eV)

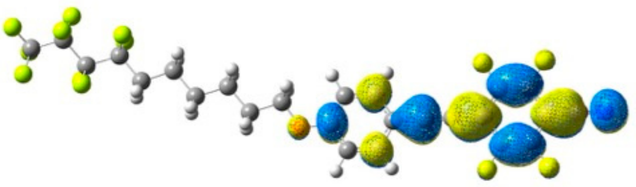

(e) Theoretical data

<Optimization>

SCF Done: E(RM062X) = –2389.37511332 Hartree A.U. after 15 cycles

Dipole moment (field-independent basis, Debye):

X= 6.6457 Y= –1.8662 Z= –0.0796 Tot= 6.9032

<TD-SCF>

Excited State 1: Singlet-A 3.6540 eV 339.31 nm f=1.6440 <S**2>=0.000

146 → 150 -0.11526

149(H) → 150(L) 0.68057

This state for optimization and/or second-order correction.

Total Energy, E(TD-HF/TD-DFT) = -2389.24083271

Copying the excited state density for this state as the 1-particle RhoCI density.

Excited State 2: Singlet-A 4.6453 eV 266.91 nm f=0.0943 <S**2>=0.000

146 → 153 -0.14106

147(H–2) → 150(L) 0.65614

149 → 153 0.16648

**Figure S23.** (a) Optimized structure of **1a** and its orbital distributions for (b) HOMO–1, (c) HOMO, and (d) LUMO. (e) Theoretical data calculated by optimization and TD-SCF calculation.

(a) Optimized structure

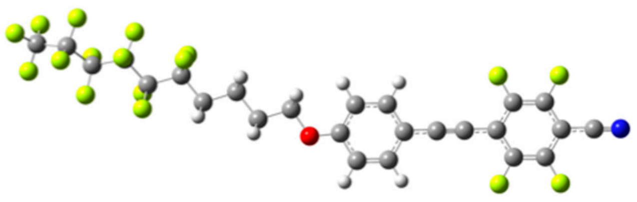

(b) HOMO–2 (–9.08 eV)

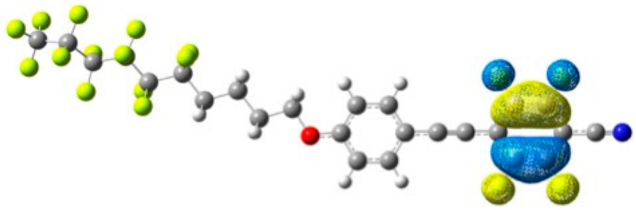

(c) HOMO (–7.52 eV)

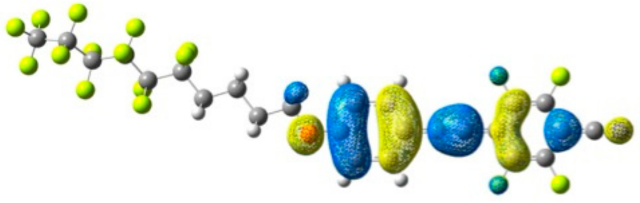

(d) LUMO (–1.97 eV)

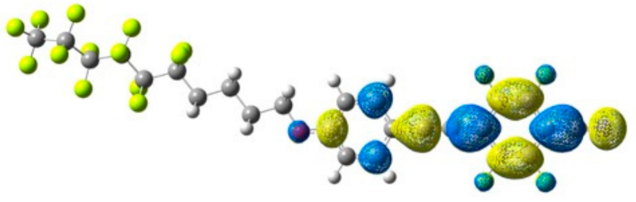

(e) Theoretical data

<Optimization>

SCF Done: E(RM062X) = –2786.21904838 Hartree A.U. after 15 cycles

Dipole moment (field-independent basis, Debye):

X= –6.5295 Y= –1.7437 Z= –0.1083 Tot= 6.7592

<TD-SCF>

Excited State 1: Singlet-A 3.6639 eV 338.40 nm f=1.6457 <S**2>=0.000

162 → 166 -0.11461

165(H) → 166(L) 0.68073

This state for optimization and/or second-order correction.

Total Energy, E(TD-HF/TD-DFT) = -2786.08440435

Copying the excited state density for this state as the 1-particle RhoCI density.

Excited State 2: Singlet-A 4.6443 eV 266.96 nm f=0.0948 <S**2>=0.000

162 → 169 -0.14122

163(H–2) → 166(L) 0.65640

165 → 169 0.16723

**Figure S24.** (a) Optimized structure of **1b** and its orbital distributions for (b) HOMO–1, (c) HOMO, and (d) LUMO. (e) Theoretical data calculated by optimization and TD-SCF calculation.

(a) Optimized structure

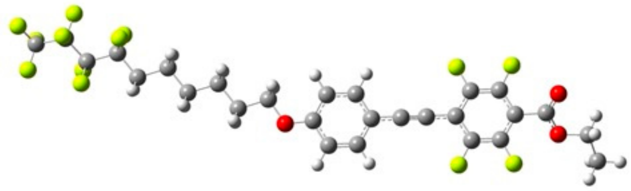

(b) HOMO–1 (–8.81 eV)

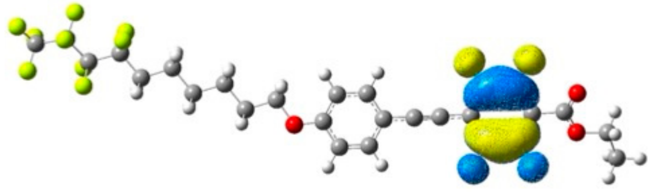

(c) HOMO (–7.43 eV)

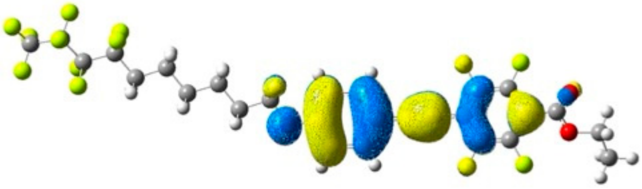

(d) LUMO (–1.65 eV)

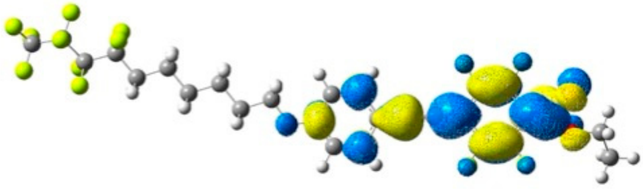

(e) Theoretical data

<Optimization>

SCF Done: E(RM062X) = -2564.26027788 Hartree A.U. after 15 cycles

Dipole moment (field-independent basis, Debye):

X= 0.7107 Y= –2.9916 Z= 1.5545 Tot= 3.4454

<TD-SCF>

Excited State 1: Singlet-A 3.8346 eV 323.33 nm f=1.6274 <S**2>=0.000

159 → 163 0.11378

162(H) → 163(L) 0.67822

This state for optimization and/or second-order correction.

Total Energy, E(TD-HF/TD-DFT) = -2564.11935728

Copying the excited state density for this state as the 1-particle RhoCI density.

Excited State 2: Singlet-A 4.6777 eV 265.05 nm f=0.0842 <S**2>=0.000

159 → 167 0.14092

161(H–1) → 163(L) 0.65489

162 → 167 0.16542

**Figure S25.** (a) Optimized structure of **2a** and its orbital distributions for (b) HOMO–1, (c) HOMO, and (d) LUMO. (e) Theoretical data calculated by optimization and TD-SCF calculation.

(a) Optimized structure

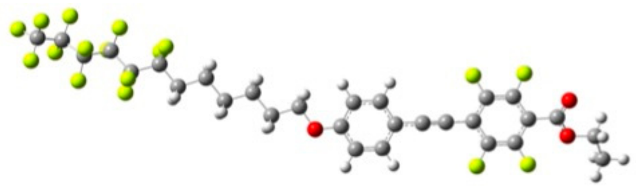

(b) HOMO–1 (–8.81 eV)

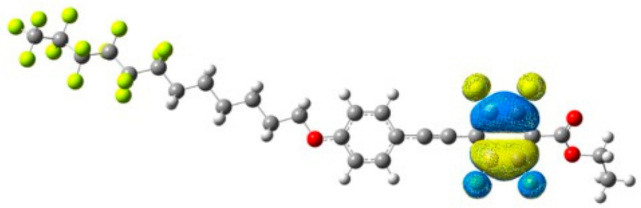

(c) HOMO (–7.43 eV)

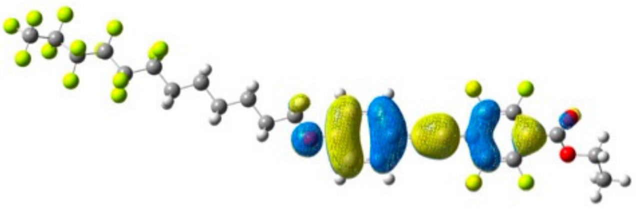

(d) LUMO (–1.65 eV)

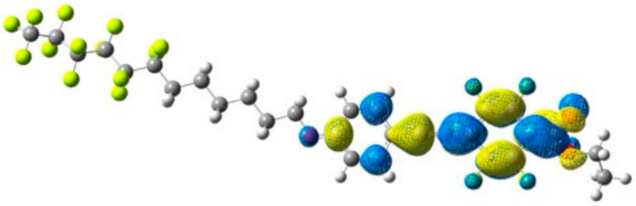

(e) Theoretical data

<Optimization>

SCF Done: E(RM062X) = -3039.69615877 Hartree A.U. after 15 cycles

Dipole moment (field-independent basis, Debye):

X= 0.6070 Y= –2.9027 Z= 1.3423 Tot= 3.2551

<TD-SCF>

Excited State 1: Singlet-A 3.8349 eV 323.30 nm f=1.6297 <S**2>=0.000

183 → 187 0.11418

186(H) → 187(L) 0.67828

This state for optimization and/or second-order correction.

Total Energy, E(TD-HF/TD-DFT) = -3039.55522848

Copying the excited state density for this state as the 1-particle RhoCI density.

Excited State 2: Singlet-A 4.6784 eV 265.02 nm f=0.0842 <S**2>=0.000

183 → 191 0.14100

185(H–1) → 187(L) 0.65477

186 → 191 0.16579

**Figure S26.** (a) Optimized structure of **2c** and its orbital distributions for (b) HOMO–1, (c) HOMO, and (d) LUMO. (e) Theoretical data calculated by optimization and TD-SCF calculation.

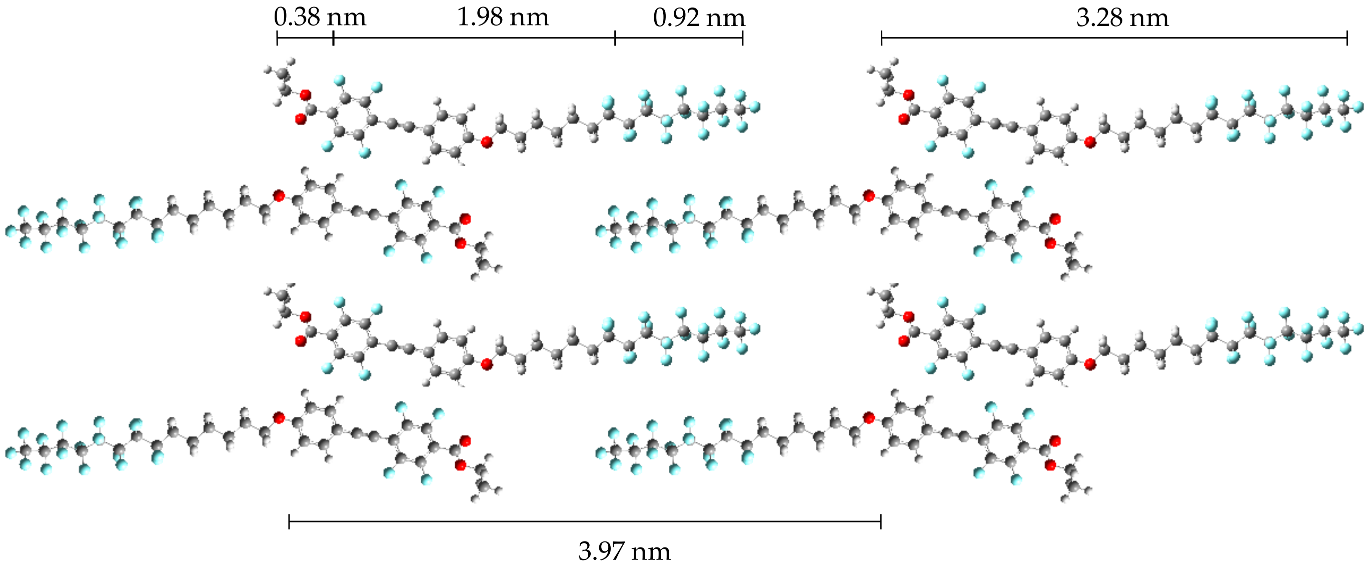


**Figure S27.** Possible aggregated structures of **2d** in the mesophase.

**Table S2.** Cartesian coordinate for **1a**.

| No. | Atom No. | Type | Coordinates (Angstroms) x | y | z |
|---|---|---|---|---|---|
| 1 | 6 | 0 | -3.260098 | -2.264895 | -0.06752 |
| 2 | 6 | 0 | -4.615129 | -1.99364 | -0.055201 |
| 3 | 6 | 0 | -5.073541 | -0.662135 | -0.023511 |
| 4 | 6 | 0 | -4.137842 | 0.380299 | -0.003811 |
| 5 | 6 | 0 | -2.77223 | 0.114458 | -0.015274 |
| 6 | 6 | 0 | -2.329259 | -1.213593 | -0.04789 |
| 7 | 1 | 0 | -2.892411 | -3.285081 | -0.092173 |
| 8 | 1 | 0 | -5.333005 | -2.807056 | -0.070208 |
| 9 | 1 | 0 | -4.483445 | 1.408574 | 0.020851 |
| 10 | 1 | 0 | -2.071649 | 0.939723 | 0.000775 |
| 11 | 6 | 0 | -6.472447 | -0.380959 | -0.011893 |
| 12 | 6 | 0 | -7.662914 | -0.147164 | -0.00253 |
| 13 | 6 | 0 | -9.055666 | 0.120643 | 0.008495 |
| 14 | 6 | 0 | -9.546467 | 1.432534 | 0.003399 |
| 15 | 6 | 0 | -9.995001 | -0.918388 | 0.02522 |
| 16 | 6 | 0 | -10.90398 | 1.693819 | 0.014279 |
| 17 | 6 | 0 | -11.353269 | -0.661669 | 0.036305 |
| 18 | 6 | 0 | -11.832449 | 0.650377 | 0.030934 |
| 19 | 8 | 0 | -1.028725 | -1.579961 | -0.063026 |
| 20 | 6 | 0 | -0.031596 | -0.558939 | -0.036581 |
| 21 | 1 | 0 | -0.156046 | 0.096673 | -0.908382 |
| 22 | 1 | 0 | -0.150037 | 0.043337 | 0.873845 |
| 23 | 6 | 0 | 1.324135 | -1.236199 | -0.061753 |
| 24 | 1 | 0 | 1.401963 | -1.841653 | -0.972545 |
| 25 | 1 | 0 | 1.394963 | -1.92029 | 0.792101 |
| 26 | 6 | 0 | 2.462935 | -0.219014 | -0.01097 |
| 27 | 1 | 0 | 2.382092 | 0.377071 | 0.907694 |
| 28 | 1 | 0 | 2.368589 | 0.48243 | -0.850616 |
| 29 | 6 | 0 | 3.837656 | -0.882727 | -0.062504 |
| 30 | 1 | 0 | 3.925744 | -1.465041 | -0.989154 |
| 31 | 1 | 0 | 3.929588 | -1.595718 | 0.767492 |
| 32 | 6 | 0 | 4.977014 | 0.132327 | 0.011956 |
| 33 | 1 | 0 | 4.900445 | 0.701276 | 0.944993 |
| 34 | 1 | 0 | 4.885536 | 0.848082 | -0.813011 |
| 35 | 6 | 0 | 6.34125 | -0.556333 | -0.059531 |
| 36 | 1 | 0 | 6.443052 | -1.116508 | -0.993521 |
| 37 | 1 | 0 | 6.463394 | -1.261927 | 0.769475 |
| 38 | 6 | 0 | 7.475878 | 0.43591 | 0.01077 |
| 39 | 6 | 0 | 8.87659 | -0.218512 | 0.013877 |
| 40 | 6 | 0 | 10.057678 | 0.756608 | -0.226239 |
| 41 | 6 | 0 | 11.435136 | 0.187767 | 0.195548 |
| 42 | 6 | 0 | -13.237066 | 0.91798 | 0.041534 |
| 43 | 7 | 0 | -14.373251 | 1.134654 | 0.049999 |
| 44 | 9 | 0 | 12.395406 | 0.976218 | -0.282222 |
| 45 | 9 | 0 | 11.542589 | 0.142337 | 1.520192 |
| 46 | 9 | 0 | 11.599275 | -1.039259 | -0.298124 |
| 47 | 9 | 0 | 9.864751 | 1.896868 | 0.460119 |
| 48 | 9 | 0 | 10.123214 | 1.042853 | -1.537725 |
| 49 | 9 | 0 | 9.05821 | -0.831995 | 1.203755 |
| 50 | 9 | 0 | 8.925255 | -1.156077 | -0.955348 |
| 51 | 9 | 0 | 7.388718 | 1.194257 | 1.137345 |
| 52 | 9 | 0 | 7.445072 | 1.297155 | -1.046964 |
| 53 | 9 | 0 | -12.211273 | -1.677425 | 0.052264 |
| 54 | 9 | 0 | -9.578986 | -2.183529 | 0.030744 |
| 55 | 9 | 0 | -8.69387 | 2.455627 | -0.012369 |
| 56 | 9 | 0 | -11.327487 | 2.954292 | 0.008771 |

**Table S3.** Cartesian coordinate for **1b**.

| No. | Atom No. | Type | Coordinates (Angstroms) x | y | z |
|---|---|---|---|---|---|
| 1 | 6 | 0 | 3.807288 | -2.231143 | 0.057314 |
| 2 | 6 | 0 | 5.163832 | -1.967287 | 0.04684 |
| 3 | 6 | 0 | 5.629431 | -0.63821 | 0.021122 |
| 4 | 6 | 0 | 4.699723 | 0.409572 | 0.007346 |
| 5 | 6 | 0 | 3.332571 | 0.151216 | 0.017962 |
| 6 | 6 | 0 | 2.882992 | -1.174431 | 0.042244 |
| 7 | 1 | 0 | 3.433802 | -3.249315 | 0.076718 |
| 8 | 1 | 0 | 5.87721 | -2.784685 | 0.058133 |
| 9 | 1 | 0 | 5.051028 | 1.435996 | -0.012055 |
| 10 | 1 | 0 | 2.636577 | 0.980507 | 0.007127 |
| 11 | 6 | 0 | 7.030045 | -0.365103 | 0.008631 |
| 12 | 6 | 0 | 8.22192 | -0.139006 | -0.002504 |
| 13 | 6 | 0 | 9.616647 | 0.118655 | -0.015366 |
| 14 | 6 | 0 | 10.117016 | 1.426665 | -0.040744 |
| 15 | 6 | 0 | 10.548236 | -0.927352 | -0.002892 |
| 16 | 6 | 0 | 11.476479 | 1.677666 | -0.052887 |
| 17 | 6 | 0 | 11.90841 | -0.680928 | -0.015039 |
| 18 | 6 | 0 | 12.397207 | 0.627339 | -0.04009 |
| 19 | 8 | 0 | 1.579743 | -1.534388 | 0.052499 |
| 20 | 6 | 0 | 0.589311 | -0.50885 | 0.035041 |
| 21 | 1 | 0 | 0.708855 | 0.132738 | 0.917848 |
| 22 | 1 | 0 | 0.713422 | 0.106718 | -0.865402 |
| 23 | 6 | 0 | -0.766713 | -1.187331 | 0.04047 |
| 24 | 1 | 0 | -0.842718 | -1.822965 | 0.929939 |
| 25 | 1 | 0 | -0.840376 | -1.839836 | -0.837057 |
| 26 | 6 | 0 | -1.899807 | -0.162371 | 0.029092 |
| 27 | 1 | 0 | -1.807933 | 0.484338 | -0.849884 |
| 28 | 1 | 0 | -1.829082 | 0.478661 | 0.914966 |
| 29 | 6 | 0 | -3.261384 | -0.859064 | 0.010688 |
| 30 | 1 | 0 | -3.366948 | -1.526446 | 0.870639 |
| 31 | 1 | 0 | -3.376109 | -1.460664 | -0.897428 |
| 32 | 6 | 0 | -4.397423 | 0.133776 | 0.056522 |
| 33 | 6 | 0 | -5.794125 | -0.517215 | -0.074897 |
| 34 | 6 | 0 | -6.982563 | 0.409486 | 0.297934 |
| 35 | 6 | 0 | -8.337416 | -0.084774 | -0.279267 |
| 36 | 6 | 0 | -9.563283 | 0.524373 | 0.4508 |
| 37 | 6 | 0 | -10.882438 | 0.412896 | -0.35395 |
| 38 | 6 | 0 | 13.803814 | 0.884361 | -0.052128 |
| 39 | 7 | 0 | 14.94158 | 1.092478 | -0.061921 |
| 40 | 9 | 0 | -11.903326 | 0.707624 | 0.445587 |
| 41 | 9 | 0 | -10.882733 | 1.259327 | -1.378372 |
| 42 | 9 | 0 | -11.033434 | -0.829567 | -0.811127 |
| 43 | 9 | 0 | -9.335418 | 1.827224 | 0.693744 |
| 44 | 9 | 0 | -9.738479 | -0.118046 | 1.61549 |
| 45 | 9 | 0 | -8.400799 | 0.248138 | -1.580891 |
| 46 | 9 | 0 | -8.40351 | -1.423222 | -0.16723 |
| 47 | 9 | 0 | -6.754156 | 1.64847 | -0.169467 |
| 48 | 9 | 0 | -7.086186 | 0.461474 | 1.639221 |
| 49 | 9 | 0 | -5.955007 | -0.928046 | -1.351408 |
| 50 | 9 | 0 | -5.848866 | -1.597563 | 0.730259 |
| 51 | 9 | 0 | -4.284956 | 1.04737 | -0.9448 |
| 52 | 9 | 0 | -4.394508 | 0.831004 | 1.22904 |
| 53 | 9 | 0 | 12.758935 | -1.702977 | -0.00246 |
| 54 | 9 | 0 | 10.122904 | -2.189151 | 0.020904 |
| 55 | 9 | 0 | 9.271919 | 2.45597 | -0.053458 |
| 56 | 9 | 0 | 11.909249 | 2.934756 | -0.076536 |

**Table S4.** Cartesian coordinate for **2a**.

| No. | Atom No. | Type | Coordinates (Angstroms) x | y | z |
|---|---|---|---|---|---|
| 1 | 6 | 0 | -2.046539 | -2.283436 | -0.135594 |
| 2 | 6 | 0 | -3.402365 | -2.013825 | -0.143562 |
| 3 | 6 | 0 | -3.863795 | -0.683748 | -0.110269 |
| 4 | 6 | 0 | -2.929648 | 0.359038 | -0.069871 |
| 5 | 6 | 0 | -1.563158 | 0.095217 | -0.062501 |
| 6 | 6 | 0 | -1.117579 | -1.231562 | -0.094923 |
| 7 | 1 | 0 | -1.677443 | -3.303164 | -0.16041 |
| 8 | 1 | 0 | -4.11848 | -2.828356 | -0.175189 |
| 9 | 1 | 0 | -3.27676 | 1.386816 | -0.044373 |
| 10 | 1 | 0 | -0.864354 | 0.921622 | -0.03165 |
| 11 | 6 | 0 | -5.26437 | -0.403836 | -0.117553 |
| 12 | 6 | 0 | -6.454478 | -0.170004 | -0.12325 |
| 13 | 6 | 0 | -7.848841 | 0.101174 | -0.130414 |
| 14 | 6 | 0 | -8.342959 | 1.405051 | -0.025687 |
| 15 | 6 | 0 | -8.792903 | -0.923968 | -0.24327 |
| 16 | 6 | 0 | -9.70214 | 1.667882 | -0.039584 |
| 17 | 6 | 0 | -10.152862 | -0.662471 | -0.243437 |
| 18 | 6 | 0 | -10.641537 | 0.640821 | -0.142978 |
| 19 | 8 | 0 | 0.184523 | -1.596331 | -0.089691 |
| 20 | 6 | 0 | 1.178547 | -0.573146 | -0.053946 |
| 21 | 1 | 0 | 1.066357 | 0.076439 | -0.932111 |
| 22 | 1 | 0 | 1.045103 | 0.035421 | 0.850161 |
| 23 | 6 | 0 | 2.536103 | -1.247276 | -0.052609 |
| 24 | 1 | 0 | 2.620437 | -1.877418 | -0.945865 |
| 25 | 1 | 0 | 2.603734 | -1.907482 | 0.820185 |
| 26 | 6 | 0 | 3.672559 | -0.226587 | -0.02511 |
| 27 | 1 | 0 | 3.570782 | 0.414549 | 0.860564 |
| 28 | 1 | 0 | 3.596515 | 0.431741 | -0.900746 |
| 29 | 6 | 0 | 5.048432 | -0.889706 | -0.011226 |
| 30 | 1 | 0 | 5.147546 | -1.541454 | -0.889258 |
| 31 | 1 | 0 | 5.131505 | -1.536567 | 0.872171 |
| 32 | 6 | 0 | 6.186442 | 0.129566 | -0.004332 |
| 33 | 1 | 0 | 6.085074 | 0.78798 | 0.865287 |
| 34 | 1 | 0 | 6.118681 | 0.760084 | -0.898344 |
| 35 | 6 | 0 | 7.550922 | -0.56127 | 0.032144 |
| 36 | 1 | 0 | 7.66306 | -1.241861 | -0.816894 |
| 37 | 1 | 0 | 7.662759 | -1.149352 | 0.949636 |
| 38 | 6 | 0 | 8.68604 | 0.431341 | -0.023 |
| 39 | 6 | 0 | 10.083823 | -0.213397 | 0.12068 |
| 40 | 6 | 0 | 11.272437 | 0.708177 | -0.255394 |
| 41 | 6 | 0 | 12.636352 | 0.226101 | 0.297596 |
| 42 | 9 | 0 | -10.982291 | -1.698353 | -0.379732 |
| 43 | 9 | 0 | -8.381774 | -2.188138 | -0.359864 |
| 44 | 9 | 0 | -7.491323 | 2.42505 | 0.094935 |
| 45 | 9 | 0 | -10.092396 | 2.936759 | 0.084159 |
| 46 | 6 | 0 | -12.103906 | 0.964919 | -0.172013 |
| 47 | 8 | 0 | -12.554744 | 1.944939 | -0.718971 |
| 48 | 8 | 0 | -12.824395 | 0.05991 | 0.477507 |
| 49 | 6 | 0 | -14.256089 | 0.270404 | 0.482924 |
| 50 | 1 | 0 | -14.457039 | 1.233978 | 0.957868 |
| 51 | 1 | 0 | -14.59765 | 0.31556 | -0.554244 |
| 52 | 6 | 0 | -14.873223 | -0.882199 | 1.240704 |
| 53 | 1 | 0 | -14.502917 | -0.912801 | 2.26803 |
| 54 | 1 | 0 | -15.958313 | -0.757887 | 1.266898 |
| 55 | 1 | 0 | -14.644388 | -1.832684 | 0.753203 |
| 56 | 9 | 0 | 12.80854 | -1.069346 | 0.035694 |
| 57 | 9 | 0 | 12.708342 | 0.418549 | 1.611487 |
| 58 | 9 | 0 | 11.373816 | 0.759289 | -1.594503 |
| 59 | 9 | 0 | 11.062489 | 1.950976 | 0.213829 |
| 60 | 9 | 0 | 10.149824 | -1.301564 | -0.674449 |
| 61 | 9 | 0 | 10.238225 | -0.617096 | 1.400673 |
| 62 | 9 | 0 | 8.685657 | 1.11519 | -1.203689 |
| 63 | 9 | 0 | 8.572202 | 1.357962 | 0.96702 |
| 64 | 9 | 0 | 13.612148 | 0.917069 | -0.28743 |

**Table S5.** Cartesian coordinate for **2c**.

| No. | Atom No. | Type | Coordinates (Angstroms) x | y | z |
|---|---|---|---|---|---|
| 1 | 6 | 0 | -4.01683 | -2.371867 | -0.099891 |
| 2 | 6 | 0 | -5.367898 | -2.079217 | -0.115029 |
| 3 | 6 | 0 | -5.806733 | -0.741296 | -0.09472 |
| 4 | 6 | 0 | -4.855143 | 0.28574 | -0.058214 |
| 5 | 6 | 0 | -3.493467 | -0.00131 | -0.042721 |
| 6 | 6 | 0 | -3.070276 | -1.335631 | -0.064219 |
| 7 | 1 | 0 | -3.665183 | -3.39792 | -0.115587 |
| 8 | 1 | 0 | -6.097637 | -2.881694 | -0.143099 |
| 9 | 1 | 0 | -5.184649 | 1.319484 | -0.042101 |
| 10 | 1 | 0 | -2.781078 | 0.813465 | -0.014395 |
| 11 | 6 | 0 | -7.202146 | -0.437188 | -0.112056 |
| 12 | 6 | 0 | -8.38769 | -0.181596 | -0.127515 |
| 13 | 6 | 0 | -9.776134 | 0.117775 | -0.146626 |
| 14 | 6 | 0 | -10.241979 | 1.435796 | -0.11582 |
| 15 | 6 | 0 | -10.742265 | -0.891524 | -0.199209 |
| 16 | 6 | 0 | -11.595281 | 1.726508 | -0.142695 |
| 17 | 6 | 0 | -12.09637 | -0.601317 | -0.211945 |
| 18 | 6 | 0 | -12.556774 | 0.715757 | -0.185107 |
| 19 | 8 | 0 | -1.774254 | -1.721601 | -0.053164 |
| 20 | 6 | 0 | -0.764697 | -0.713591 | -0.021836 |
| 21 | 1 | 0 | -0.869895 | -0.063295 | -0.900287 |
| 22 | 1 | 0 | -0.886331 | -0.102135 | 0.882046 |
| 23 | 6 | 0 | 0.583314 | -1.406581 | -0.025229 |
| 24 | 1 | 0 | 0.659035 | -2.031573 | -0.922897 |
| 25 | 1 | 0 | 0.642623 | -2.073469 | 0.843025 |
| 26 | 6 | 0 | 1.732467 | -0.400346 | 0.009033 |
| 27 | 1 | 0 | 1.647002 | 0.22515 | 0.907544 |
| 28 | 1 | 0 | 1.655472 | 0.275279 | -0.853232 |
| 29 | 6 | 0 | 3.100937 | -1.078631 | -0.004365 |
| 30 | 1 | 0 | 3.189483 | -1.701234 | -0.904498 |
| 31 | 1 | 0 | 3.180671 | -1.755801 | 0.856217 |
| 32 | 6 | 0 | 4.249278 | -0.071604 | 0.031728 |
| 33 | 1 | 0 | 4.173198 | 0.53907 | 0.938735 |
| 34 | 1 | 0 | 4.168622 | 0.607426 | -0.824082 |
| 35 | 6 | 0 | 5.607544 | -0.774946 | 0.001415 |
| 36 | 1 | 0 | 5.721912 | -1.355805 | -0.920407 |
| 37 | 1 | 0 | 5.706022 | -1.463847 | 0.845336 |
| 38 | 6 | 0 | 6.750739 | 0.207541 | 0.073078 |
| 39 | 6 | 0 | 8.144693 | -0.449128 | -0.059421 |
| 40 | 6 | 0 | 9.336198 | 0.465255 | 0.334114 |
| 41 | 6 | 0 | 10.691651 | -0.026663 | -0.243367 |
| 42 | 9 | 0 | -12.948074 | -1.625373 | -0.286722 |
| 43 | 9 | 0 | -10.358521 | -2.168841 | -0.243228 |
| 44 | 9 | 0 | -9.3685 | 2.442626 | -0.055587 |
| 45 | 9 | 0 | -11.957895 | 3.008549 | -0.09233 |
| 46 | 6 | 0 | -14.012115 | 1.069224 | -0.227183 |
| 47 | 8 | 0 | -14.444965 | 2.024593 | -0.829478 |
| 48 | 8 | 0 | -14.747942 | 0.220309 | 0.478434 |
| 49 | 6 | 0 | -16.174813 | 0.461364 | 0.478164 |
| 50 | 1 | 0 | -16.35261 | 1.454909 | 0.897409 |
| 51 | 1 | 0 | -16.521513 | 0.453543 | -0.558253 |
| 52 | 6 | 0 | -16.811411 | -0.631969 | 1.304565 |
| 53 | 1 | 0 | -16.435221 | -0.611121 | 2.329999 |
| 54 | 1 | 0 | -17.893437 | -0.48307 | 1.329174 |
| 55 | 1 | 0 | -16.605896 | -1.613754 | 0.871747 |
| 56 | 6 | 0 | 11.916731 | 0.566583 | 0.501212 |
| 57 | 6 | 0 | 13.238548 | 0.462414 | -0.30018 |
| 58 | 9 | 0 | 13.24708 | 1.326456 | -1.309736 |
| 59 | 9 | 0 | 13.385156 | -0.772656 | -0.778395 |
| 60 | 9 | 0 | 14.257912 | 0.737957 | 0.508199 |
| 61 | 9 | 0 | 11.693206 | 1.866408 | 0.763668 |
| 62 | 9 | 0 | 10.763262 | 0.324365 | -1.539871 |
| 63 | 9 | 0 | 10.751534 | -1.366853 | -0.150032 |
| 64 | 9 | 0 | 9.11756 | 1.712968 | -0.11446 |
| 65 | 9 | 0 | 9.433434 | 0.495363 | 1.676675 |
| 66 | 9 | 0 | 8.311664 | -0.842415 | -1.340834 |
| 67 | 9 | 0 | 8.189605 | -1.541613 | 0.730043 |
| 68 | 9 | 0 | 6.652941 | 1.139603 | -0.913176 |
| 69 | 9 | 0 | 6.745498 | 0.884889 | 1.257657 |
| 70 | 9 | 0 | 12.08483 | -0.09447 | 1.656519 |
